# A multiplex single-cell RNA-Seq pharmacotranscriptomics pipeline for drug discovery

**DOI:** 10.1038/s41589-024-01761-8

**Published:** 2024-10-31

**Authors:** Alice Dini, Harlan Barker, Emilia Piki, Subodh Sharma, Juuli Raivola, Astrid Murumägi, Daniela Ungureanu

**Affiliations:** 1 Disease Networks Unit, Faculty of Biochemistry and Molecular Medicine, University of Oulu, Oulu, Finland; 2 Tampere University Hospital and Faculty of Medicine and Health Technology, Tampere University, Tampere, Finland; 3 Applied Tumor Genomics, Research Program Unit, Faculty of Medicine, University of Helsinki, Helsinki, Finland; 4 Institute for Molecular Medicine Finland (FIMM), Helsinki Institute of Life Science (HiLIFE), University of Helsinki, Helsinki, Finland

**Keywords:** Phenotypic screening, Cancer therapy, High-throughput screening, High-throughput screening

## Abstract

The gene-regulatory dynamics governing drug responses in cancer are yet to be fully understood. Here, we report a pipeline capable of producing high-throughput pharmacotranscriptomic profiling through live-cell barcoding using antibody–oligonucleotide conjugates. This pipeline combines drug screening with 96-plex single-cell RNA sequencing. We show the potential of this approach by exploring the heterogeneous transcriptional landscape of primary high-grade serous ovarian cancer (HGSOC) cells after treatment with 45 drugs, with 13 distinct classes of mechanisms of action. A subset of phosphatidylinositol 3-OH kinase (PI3K), protein kinase B (AKT) and mammalian target of rapamycin (mTOR) inhibitors induced the activation of receptor tyrosine kinases, such as the epithelial growth factor receptor (EGFR), and this was mediated by the upregulation of caveolin 1 (CAV1). This drug resistance feedback loop could be mitigated by the synergistic action of agents targeting PI3K–AKT–mTOR and EGFR for HGSOC with CAV1 and EGFR expression. Using this workflow could enable the personalized testing of patient-derived tumor samples at single-cell resolution.

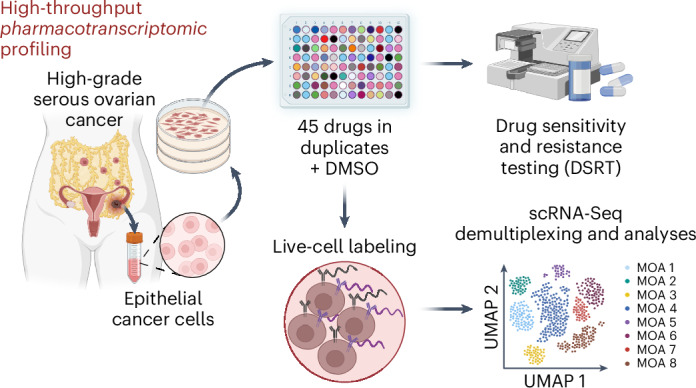

## Abstract (web summary)

A high-throughput precision oncology pipeline for pharmacotranscriptomic profiling using live-cell barcoding and single-cell RNA sequencing was developed. Using this approach revealed a drug resistance feedback loop in high-grade serous ovarian cancer, suggesting that combining phosphatidylinositol 3-OH kinase, protein kinase B and mammalian target of rapamycin inhibitors with epithelial growth factor receptor inhibitors may overcome resistance.

## Main

Large-scale conventional transcriptomics has been used to produce extensive datasets of perturbagen signatures, most notably the Library of Integrated Network-Based Cellular Signatures (LINCS) dataset^1–5^, which provides unprecedented insights into the molecular networks of cancer phenotypes. Often, it is the ‘switching on–off’ and ‘wiring–rewiring’ of these networks that determine the drug responses of cancer cells, involving complex signaling circuitry and nongenetic drivers of drug sensitivity. However, a key bottleneck in overcoming drug resistance has been the notable variability in drug responses because of cancer heterogeneity imposing genetic, transcriptomic, epigenetic and/or phenotypic changes at the level of individual patient cells^6–9^. Recent methods using single-cell RNA sequencing (scRNA-Seq) with combinatorial barcoding have enabled the efficient study of transcriptional signatures after pharmacological perturbation of cell lines^1,10–14^. Implementing such technologies using tumor-derived primary cancer cells ex vivo could not only uncover the extent of cancer heterogeneity at the single-cell level but also provide an accurate assessment of drug response mechanisms, which could greatly advance the development of more effective personalized therapies^9^. Yet, the current cost of scRNA-Seq technologies and the limited availability of clinical samples are challenges to be overcome.

Here, we applied a transcriptome-based precision oncology pipeline for the systematic identification of single-cell transcriptomic responses to drugs of cancer cells through multiplexed scRNA-Seq powered by combinatorial barcoding of live epithelial cancer cells from ex vivo cultures of primary samples. We illustrate the feasibility of our pipeline by investigating the transcriptomic landscape of drug responses in high-grade serous ovarian cancer (HGSOC), the most common ovarian cancer (OC) subtype, which accounts for 70–80% of all OC cases^15–18^. Interpatient and intrapatient tumor heterogeneity is one of the main obstacles to HGSOC therapeutic success, supported by strong patterns of chromosomal instability across tumors and/or throughout metastatic sites^19–22^. Initial clinical management for patients with HGSOC involves debulking surgery followed by platinum-based and/or taxane-based chemotherapy, with some patients receiving poly(ADP-ribose) polymerase (PARP) inhibitors^23,24^. However, relapse develops in almost 80% of HGSOCs, at which point treatment options are mostly palliative. This highlights an unmet need for improving the therapeutic outcome of this deadly malignancy.

In our study we (1) used 96-plex scRNA-Seq through antibody-conjugate-mediated barcoding of cancer cells treated with 45 drugs covering 13 mechanisms of action (MOAs) defining the drug pharmacological effect, across a wide range of therapeutic targets; (2) screened three HGSOC models, including two ex vivo patient-derived tumor epithelial cancer cells (PDCs) representing clinically distinct disease stages; (3) integrated the transcriptomic profiles of 288 treated samples at a single-cell level across ~36,000 cells; and (4) validated the most relevant findings, such as a previously unobserved phosphatidylinositol 3-OH kinase (PI3K), protein kinase B (AKT) and mammalian target of rapamycin (mTOR) inhibitor-driven drug resistance mechanism resulting from a caveola signaling feedback loop in HGSOC. Our study outlines an integrative pharmacotranscriptomic screen for elucidating target genes and pathways governing drug responses in cancer cells. As a result, we generated a rich resource for investigating drug-perturbed phenotypes at the single-cell level for the identification of therapeutic options in HGSOC in a cost-efficient and time-efficient manner, which could also be successfully applied to other cancers.

## Results

### A 96-plex scRNA-Seq pipeline for drug responses in HGSOC

**Fig. 1:**
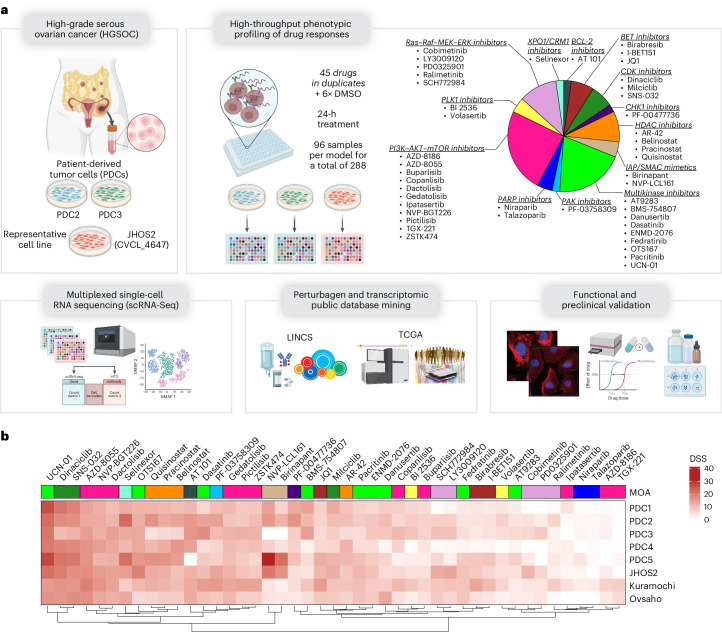
Graphical abstract of the experimental setup. **a**, Experimental setup for multiplexed scRNA-Seq. The representative HGSOC models (PDC2, PDC3 and JHOS2) underwent 96-plex scRNA-Seq after 24 h of treatment with 45 drugs from 13 MOAs, reported in the pie chart on the right, or DMSO as a control. Results were further computationally and experimentally validated using the LINCS database. **b**, Heat map depicting the drug sensitivity profile as DSSs from five PDCs and three cell lines representing HGSOC. The DSSs were clustered using complete-linkage hierarchical clustering based on Euclidean distances. Color bars indicate MOAs. The HGSOC preclinical models that underwent 96-plex scRNA-Seq are highlighted in bold. Source data

To understand the heterogeneity of drug responses in HGSOC, we set up a precision oncology pipeline combining high-throughput drug testing with 96-plex scRNA-Seq of individual drug treatments (Fig. 1a, Supplementary Fig. 1a and Methods). First, we performed a drug sensitivity and resistance testing (DSRT) screen using three representative HGSOC cell lines (JHOS2, Kuramochi and Ovsaho) and five HGSOC PDCs (from primary, postneoadjuvant chemotherapy (NACT) or relapsed cases; Supplementary Fig. 1b). The PDCs were isolated and cultured ex vivo at early passages to avoid loss of phenotypic identity^25^.

DSRT was carried out using a library of 45 drugs from 13 distinct pharmacological classes as defined by their MOAs (Fig. 1a,b). The drug library included investigational and approved compounds used in cancer treatments such as kinase inhibitors targeting the PI3K–AKT–mTOR pathway that is frequently dysregulated in OC, the Ras–Raf–MEK (mitogen-activated protein kinase kinase) pathway modulating signaling downstream growth factors by receptor tyrosine kinases (RTKs), inhibitors of polo-like kinase (PLK), cyclin-dependent kinase (CDK), and checkpoint kinase (CHK) involved in the modulation of cell-cycle machinery, epigenetic modifiers (inhibitors of histone deacetylase (HDAC) and bromodomain and extraterminal domain (BET)) regulating chromatin integrity, DNA damage proteins (PARP inhibitors) regulating homologous recombination (HR)-related processes and apoptotic modulators (B cell lymphoma 2 (BCL-2) inhibitors, inhibitor of apoptosis (IAP) and second mitochondrial-derived activator of caspases (SMAC) mimetics and inhibitors of exportin 1) involved in cell death regulation. The drug responses were evaluated using bulk cell viability assays as a drug sensitivity score (DSS), which captures and integrates the dose–response curve (10,000-fold dilution range) into a single metric^26^. The cutoff for a significant drug response was defined by a DSS value of 12.2, representing the 75th percentile of the DSS distribution of all drugs across all samples (Supplementary Fig. 1c). Targeted agents such as inhibitors of PI3K–AKT–mTOR, Ras–Raf–MEK–ERK (extracellular signal-regulated kinase), CDK and HDAC showed good but variable ex vivo efficacy across cell lines and PDCs. However, understanding drug MOAs is challenging from the genomic background and cell viability screens alone.

### Transcriptomic landscape of drug responses in HGSOC

To investigate the transcriptional landscape withstanding drug treatment in HGSOC, we proceeded with 96-plex scRNA-Seq using Cell Hashing^12^ of three HGSOC samples: JHOS2 (a representative cell line), PDC2 and PDC3 (ovary primary tissue samples from post-NACT cases). The HGSOC cells were treated for 24 h with 45 drugs (DMSO as control) in duplicates using a drug concentration above the half-maximal effective concentration based on DSRT screens, which was likely able to elicit a transcriptional response across all drugs (Supplementary Table 1). Following drug treatments, cells in each well were labeled with a unique pair of anti-β_2_ microglobulin (B2M) and anti-CD298 antibody–oligo conjugates or Hashtag oligos (HTOs) from a set of 20 (12 for the columns and 8 for the rows of a 96-well plate) before sample pooling for multiplexed scRNA-Seq (Methods and Supplementary Fig. 1a). Following preprocessing, we demultiplexed the transcriptomic profile of 36,016 high-quality cells over a total of 288 samples, with a median of 140 cells per well for JHOS2 and 122 cells per well for both PDC2 and PDC3 (Supplementary Fig. 2a). The percentage of successfully retained cells with double-HTO labeling slightly varied across the three models but ranged between 40% and 50%, which can be attributed to the variable expression of *CD298* along with the effect of the compounds on the HTO–antibody conjugates targeting the *B2M* and *CD298* genes (Supplementary Fig. 2b).

**Fig. 2:**
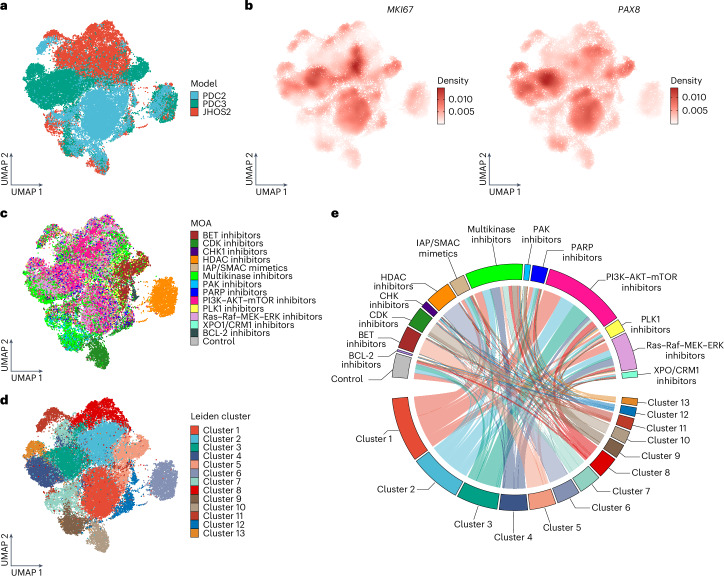
The transcriptomic landscape of HGSOC drug responses. **a**, UMAP embedding of 36,016 treated HGSOC cells from PDC2, PDC3 and JHOS2, colored by model of origin. **b**, Gene expression of *PAX8* and *MKI67*. Colors refer to gene-weighted kernel density. **c**,**d**, Same UMAP plots as in (**a**) but colored by the drug MOAs used for cell treatment (**c**) or by assigned Leiden cluster (**d**). **e**, Chord diagram showing cell distribution by MOAs across Leiden clusters, with arch thickness indicating cell proportion.

The uniform manifold approximation and projection (UMAP) embedding highlighted both the separation and the convergence of the three samples (Fig. 2a). The epithelial OC origin of the PDC2 and PDC3 samples was confirmed by the expression of OC-specific markers *PAX8* and *CD24*, epithelial makers *MKI67, EPCAM, KRT8* and *KRT18* and cancer stem cell markers such as *CD44* and *ROR1* (Fig. 2b and Supplementary Fig. 2c). Moreover, various drug treatments induced *MKI67* and *PAX8* gene expression variation (Fig. 2b), as observed with *B2M* and *CD298* genes. Next, our analysis incorporated Leiden clustering to find cells with similar transcriptional profiles within the dataset. Interestingly, while the main mass of cells in the three models retained separation consistent with their overlying phenotypic and genomic differences, Leiden clustering identified 13 clusters with homogeneous or heterogeneous composition of cells coming from (1) different MOAs but the same models (clusters 1, 2, 3, 4 and 8); (2) same MOAs but different models (clusters 5, 6, 9, 10 and 12); or (3) different MOAs and models (clusters 7, 9, 11 and 13) (Fig. 2c,d). Overall, we observed that cells treated with PI3K–AKT–mTOR, Ras–Raf–MEK–ERK and multikinase inhibitors were primarily observed as cases 1 and 3, suggesting that cells treated with these intracellular signaling inhibitors underwent a milder and model-specific transcriptional shift. On the other hand, cells treated with BET, HDAC and CDK inhibitors were mostly found in distinct clusters enriched with cells from all the three models (Fig. 2e and Supplementary Fig. 2d). The distribution of cell-cycle phases was heterogeneous across all clusters and treatments (Supplementary Fig. 2e).

**Extended Data Fig. 1:**
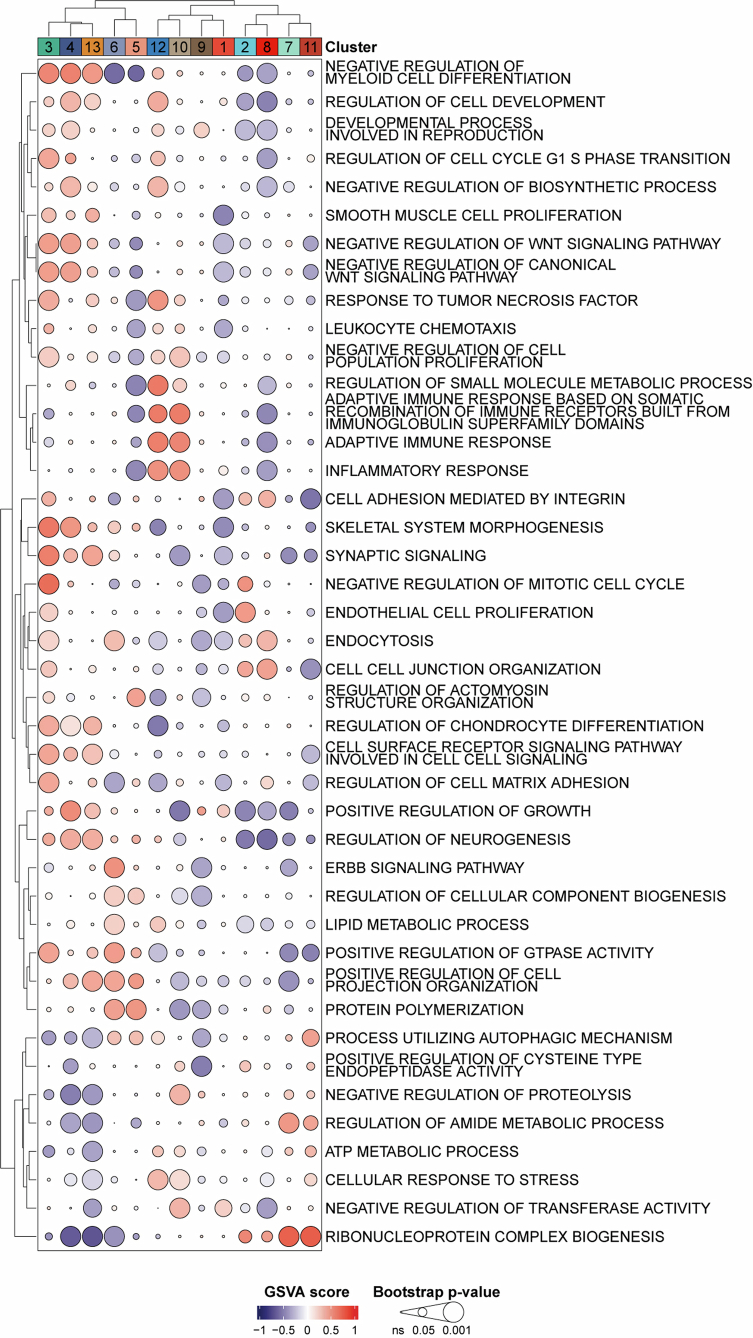
Gene ontology biological process activity in clusters using gene set variation analysis (GSVA). GSVA scores of top 50 positive and negative markers of each Leiden cluster for the 5% most variable gene ontology biological processes (GO:BP). Color scale indicates the enrichment and directionality, circle size denotes the one-sided *p*-value computed via bootstrapping. Source data

Gene set variation analysis (GSVA) was carried out to evaluate the activity of biological processes defined by gene ontology (GO:BP) within each of the clusters (Extended Data Fig. 1). Our analysis showed that clusters 7 and 11, composed of cells treated with various kinase inhibitors, had a lower GSVA score in terms related to the modulation of guanosine triphosphatase (GTPase) activity and signaling and positive scores for ribonucleoprotein complex biogenesis and metabolism, in accordance with their role in regulating intracellular signaling. Furthermore, processes including the negative regulation of catalytic activity, molecular function and viral responses were enriched in clusters 10 and 12 comprising mostly cells treated with CDK and BCL-2 inhibitors. Cells treated with BET inhibitors were found mainly in cluster 5, where GO terms indicated the downregulation of various inflammatory responses and upregulation of GTPase activity and signaling and membrane and protein assembly or complexes. Similarly, cells treated with HDAC inhibitors mapped mainly to cluster 6, which showed enrichment in GO:BPs related to endocytosis, ErbB signaling, GTPase activity and protein polymerization. Clusters 3, 4 and 13 were primarily composed of PDC3 cells treated with drugs from various MOAs. These three clusters elicited a similar trend in their GSVA scores, with positive terms for processes related to receptor signaling, negative canonical Wnt signaling, muscle and epithelium development and regulation of GTPases and the cell cycle, suggesting that these pathways are actively modulated by drugs in PDC3.

### MOA-specific transcriptomic changes in HGSOC

**Fig. 3:**
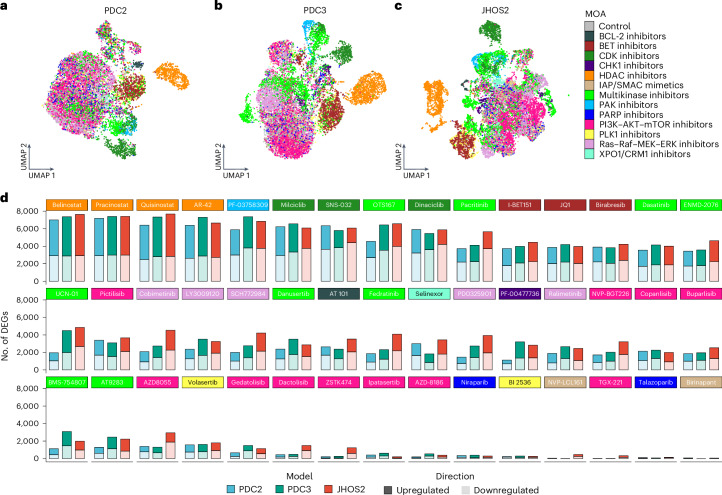
The model-specific transcriptome modulation by targeted drugs in HGSOC. **a–c**, UMAP plots of HGSOC models PDC2 (**a**), PDC3 (**b**) and JHOS2 (**c**), with colors corresponding to the drug MOAs. **d**, Bar charts illustrating the number of differentially expressed protein-coding genes (|log_2_FC| > 0.5, FDR < 0.01) observed in the comparison of subsample pseudobulk aggregates of treated versus untreated cells. The color-coded headers correspond to drug MOAs indicated in **a**–**c**. Source data

To better address both the heterogeneity and the convergence in drug responses of each model, we projected their cells in three model-specific UMAPs (Fig. 3a–c). Following the previous results, cells from the three models showed a similar spatial distribution of MOAs, retaining the distinct clusters of cells treated with HDAC, BET and CDK inhibitors observed in the integrated data.

To evaluate the extent of the transcriptomic changes elicited by each of the 45 drugs, we computed subsamples followed by pseudobulk aggregation (summing gene counts from multiple cells into a single value for each gene) of each treatment group (drug and model; Supplementary Fig. 3a and Methods). Combining subsampling and aggregation (sum)-based pseudobulk offered a parallel computational strategy to validate high-level findings while enhancing the reproducibility and robustness of differential gene expression (DGE) analyses of the limited sample sizes and sparsity characteristic of scRNA-Seq. We performed DGE analysis between pseudobulk aggregates of drug-treated versus control (DMSO) cell subsets, where the number of differentially expressed genes obtained in each comparison could serve as a proxy measure of the magnitude of transcriptional changes elicited by each compound (Supplementary Fig. 3a). The three HGSOC models showcased distinct patterns of upregulated and downregulated protein-coding genes (|log_2_ fold change (log_2_FC)| > 0.5, false discovery rate (FDR) < 0.01), yet cells treated with drugs associated with the same MOA displayed similar patterns within the same model (Fig. 3d). Although the observed changes in transcription for some drugs were smaller compared to others (for example, PI3K–AKT–mTOR inhibitors (AZD-8186, ipatasertib and ZSTK474), PARP inhibitors (niraparib and talazoparib), SMAC mimetics (birinapant and NVP-LCL161) and PLK1 inhibitors (BI 2536)), their efficacy was not directly correlated to their overall transcriptional dynamics, as previously demonstrated (Fig. 1b)^27^.

For the majority of the compounds, a similar shift in transcription was noticeable among the three models. Moreover, the number of downregulated genes detected after treatment with the BCL-2 inhibitor AT-101 appeared lower in PDC2 compared to PDC3 and JHOS2 (Fig. 3d). To investigate the signaling dynamics perturbed by the different classes of drugs, we submitted the union of the significantly upregulated or downregulated genes from all drugs in each of the MOAs to over-representation analysis (ORA) against the Reactome database^28^ (Supplementary Fig. 3b and Methods). To this aim, we filtered the resulting biological processes for Reactome pathways involved in cancer development, progression and therapeutics (Supplementary Table 2). Different MOAs were sometimes associated with overlapping signaling perturbations, suggesting that different drugs can lead to both convergent and divergent phenotypes. In particular, while some pathways appeared ubiquitously over-represented in the three models, others were MOA or model specific, validating the observations derived from the previous scRNA-Seq results. For instance, HR repair pathways were reported as significantly enriched (FDR < 0.05) for the downregulated genes across nearly all the MOAs, whereas genes upregulated in the PI3K–AKT–mTOR inhibitor MOA showed enrichment for forkhead box O (FOXO) signaling in all three models (Supplementary Fig. 3b).

In line with our DGE analysis, MOAs reporting similar trends between JHOS2 and PDC2 likewise possessed an enrichment of the same Reactome pathway processes in these two models (Supplementary Fig. 3b). Specifically, only JHOS2 and PDC2 reported an enrichment of intracellular signaling by FOXO and Notch among upregulated genes in cells treated with BCL-2 inhibitors and more pronounced downregulation of HR genes in cells treated with CDK, HDAC, p21-activated kinase and PI3K–AKT–mTOR inhibitors. Lastly, a specific cluster of pathways (FOXO-mediated transcription, signaling by Notch and FOXO-mediated transcription of cell-cycle genes) stood out as being enriched in the upregulated genes of JHOS2 and PDC2 cells treated with PI3K–AKT–mTOR inhibitors. Moreover, signaling by platelet-derived growth factor receptor and ErbB2 was enriched in upregulated genes in JHOS2 and PDC2 cells with BET inhibitor treatment. This suggests that compensatory feedback loops elicited by these compounds might fuel drug resistance mechanisms. On the basis of these joint profiles, our results indicate that JHOS2 and PDC2 harbor a similar transcriptional regulation of their drug responses, particularly for certain MOAs such as PI3K–AKT–mTOR, HDAC, BET and CDK inhibitors.

### PI3K–AKT–mTOR inhibitors mediated drug responses in HGSOC

One of the pathways frequently altered in OC is PI3K–AKT–mTOR, with 45% of cases presenting PI3K signaling aberrations such as amplification of *PIK3CA*, *AKT* isoforms (*AKT2* and *AKT3*) or *RICTOR* and deletion of *PTEN*^29^. Recent molecular profiling data of more than 200 HGSOC clinical samples showed that the PI3K–AKT pathway remained activated despite chemotherapy treatment or disease progression, suggesting that a pharmacological intervention with targeted inhibitors would be of great benefit^30^. However, the genomic complexity of HGSOC tumors and the intolerable side effects of PI3K–AKT–mTOR inhibitors have so far led to disappointing results in clinical trials, especially as monotherapy^31^. Only alpelisib, selective for the PI3Kα isoform, has received US Food and Drug Administration (FDA) approval for solid tumors such as breast cancer, in combination with the FDA-approved endocrine therapy fulvestrant^32^.

Our PI3K–AKT–mTOR library included 11 inhibitors with various substrate specificities such as pan-PI3K inhibitors (AZD-8186, buparlisib, and pictilisib), adenosine triphosphate-competitive pan-PI3K, AKT and mTOR inhibitors (copanlisib, ipatasertib, ZSTK474 and AZD-8055), dual PI3K and mTOR inhibitors (dactolisib, gedatolisib and NVP-BGT226) and specific PI3Kβ inhibitor TGX-221 (Supplementary Fig. 4a). However, as expected and observed in our DSRT screen, the efficacies of these drugs did not follow a clear substrate specificity or pharmacological efficacy across HGSOC cell lines and PDCs (Fig. 1b).

When projecting cells from the three models into three-dimensional (3D) UMAPs, we could resolve a clear overlap of untreated JHOS2 and PDC3 cells, whereas, in cells treated, for instance, with PI3K–AKT–mTOR inhibitors AZD-8055, buparlisib, gedatolisib and NVP-BGT226, JHOS2 cells shifted closer to or even intersected with PDC2 cells (Supplementary Fig. 4b). The clear distinction among PDC3 and PDC2 clusters for both untreated cells and those treated with PI3K–AKT–mTOR inhibitors may reflect intrapatient heterogeneity, as both PDC2 and PDC3 were collected from ovarian tissue at the interval stage (post-NACT). JHOS2 cells are documented to originate from a metastatic lymph node of a person with HGSOC but the clinical progression stage is unknown^33,34^.

**Fig. 4:**
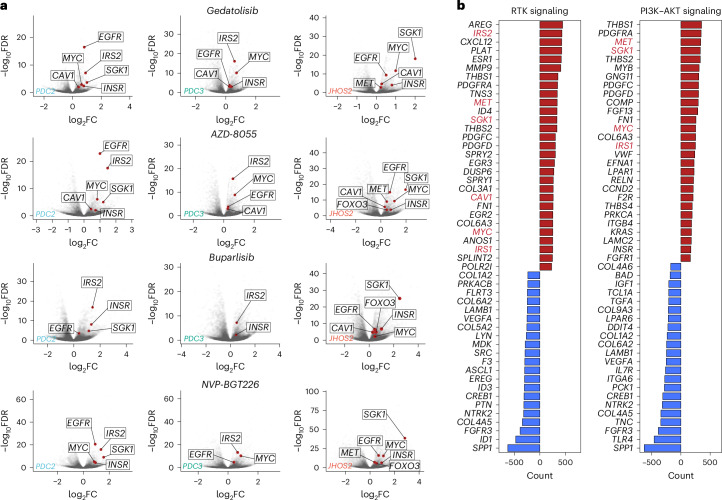
Multiplexed scRNA-Seq captures PI3K–AKT inhibitor resistance feedback loops. **a**, Volcano plots reporting the distribution of the gene expression FCs and *P* values for the comparisons of subsample pseudobulk aggregates of treated versus untreated cells for the indicated PI3K–AKT–mTOR inhibitors. Only statistically significant, differentially expressed genes (FDR < 0.01) are labeled. **b**, Waterfall plots depict the total counts of occurrence for genes characteristic of RTK or PI3K–AKT signaling, as defined in MSigDB C2 curated gene sets, in OC and breast cancer cell lines treated with PI3K–AKT inhibitors from the LINCS database. Positive values indicate the count of occurrence in upregulated gene sets in LINCS perturbation experiments, while negative values indicate the count of occurrence in downregulated gene sets.

When we delved into the results of the DGE analyses of cells treated with the aforementioned PI3K–AKT–mTOR inhibitors versus untreated cells, we discovered a similar modulation of RTKs such as *MET*, the metabolic driver *MYC* and insulin signaling-related genes (*IRS1*, *IRS2*, *INSR* and *SGK1*), together with caveolin 1 (*CAV1*) (Fig. 4a). Interestingly, *MET* and *CAV1* share a close genomic neighborhood on chromosome 7 where *PIK3CG*, *SERPINE1* and *EGFR* genes are also located, indicating a possible common transcriptional regulation among these genes (Supplementary Fig. 4c). Specifically, *CAV1*, *MET*, *MYC*, *IRS1*, *IRS2*, *INSR* and *SGK1* were frequently upregulated in all three models. Interestingly, other genes that followed the same trend included *FOXO3*, known to promote the transcription of RTKs and reactivation of the signaling cascade in response to AKT inhibitor treatment^35^, and *EGFR*, a direct interaction partner of CAV1 (refs. ^36,37^). To validate our findings, we interrogated the LINCS dataset for the most frequently upregulated and downregulated genes in OC and breast cancer (genomically related cancers driven by copy-number aberration and hormone regulation) cell lines treated with PI3K–AKT inhibitors (*n* = 34; Methods). *MET*, *MYC* and insulin signaling-related genes (*IRS1*, *IRS2*, *INSR* and *SGK1*) were identified among the most upregulated genes, together with *CAV1* (Fig. 4b).

### CAV1–EGFR interplay in HGSOC treatment and progression

**Fig. 5:**
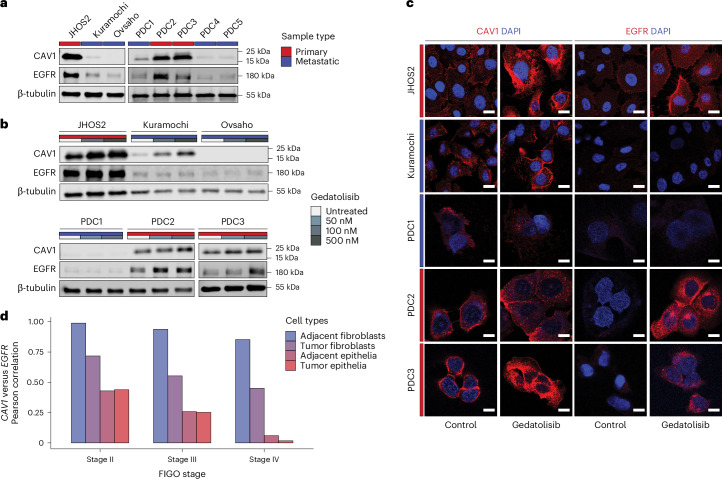
PI3K–AKT–mTOR inhibitors enhance the protein levels of CAV1 and EGFR in subsets of HGSOC cells. **a**,**b**, Immunoblots showing the protein levels of CAV1 and EGFR in HGSOC models according to sample type (**a**) and gedatolisib treatment (**b**). β-tubulin was used as a loading control. For each of the immunoblots, a representative of three independent experiments is shown. Gedatolisib treatment was applied for 48 h using the indicated concentrations. **c**, Immunofluorescence staining of CAV1 or EGFR (in red) and DAPI (in blue) of HGSOC models after 48-h treatment with gedatolisib using the same concentrations as in **b**. Scale bar, 20 µm. A representative of three independent experiments is shown. **d**, Pearson correlation of *CAV1* and *EGFR* mRNA levels from the deconvoluted TCGA data of HGSOC samples (*n* = 429) based on FIGO stages II, III and IV. Source data

Our analyses identified *CAV1* and *EGFR* as genes differentially modulated by PI3K–AKT–mTOR inhibitors in our HGSOC preclinical models. To validate these findings, we assessed CAV1 and EGFR protein expression in our HGSOC models by immunoblotting, which revealed a strong expression of both proteins in JHOS2, PDC2 and PDC3 but not in PDC1, PDC4, PDC5, Kuramochi and Ovsaho, which are all metastatic samples derived from ascites or pleural effusion (Fig. 5a). Moreover, CAV1 coimmunoprecipitated with EGFR in JHOS2 cells, indicative of a direct interaction between these proteins, as previously observed in other cancers (Supplementary Fig. 5a)^36,38^.

We then addressed the question of whether PI3K–AKT–mTOR inhibitors can modulate CAV1 and EGFR protein levels in HGSOC models as indicated by our transcriptomic analyses (Fig. 4a). Both immunoblotting and immunofluorescence analyses showed that, after 48 h of treatment, the dual PI3K–mTOR inhibitor gedatolisib induced a dose-dependent and variable increase in CAV1 and EGFR protein levels in JHOS2, PDC2 and PDC3 cells but not in PDC1 and Ovsaho cells, which lack CAV1 expression (Fig. 5b,c). To assess whether CAV1 directly modulates EGFR expression in OC cells, we overexpressed CAV1-Flag followed by gedatolisib treatment to monitor changes in EGFR levels in JHOS2 and Kuramochi cells. Immunoblotting analyses showed that CAV1 overexpression enhanced EGFR levels in JHOS2 but not in Kuramochi cells, which do not express EGFR, and this effect was augmented by gedatolisib treatment (Supplementary Fig. 5b). Next, we assessed the effect of CAV1 or EGFR knockdown by small interfering RNA (siRNA) on the gedatolisib-induced upregulation of CAV1 and EGFR in JHOS2 cells. Immunoblotting analysis showed that knockdown of either CAV1 or EGFR impaired gedatolisib-mediated upregulation of EGFR or CAV1, respectively, indicating that both CAV1 and EGFR expression is needed for the full effect of gedatolisib-induced upregulation of either protein (Supplementary Fig. 5c). Interestingly, although gedatolisib could slightly upregulate CAV1 protein levels in Kuramochi cells, EGFR expression was not induced, which would suggest a cell-specific regulatory mechanism of CAV1 signaling in response to PI3K–mTOR inhibition, likely influenced by *KRAS* amplification^39^ in these cells. Moreover, phosphorylated (p)AKT, pERK1 and pERK2 levels were not affected by gedatolisib in any of the HGSOC models except Ovsaho, although pAKT was inhibited in all cell lines upon 500 nM gedatolisib treatment (Supplementary Fig. 5d). Taken together, our results demonstrate that targeted inhibition of PI3K–mTOR signaling with gedatolisib induces a feedback regulatory loop of RTK upregulation through EGFR that is dependent on both CAV1 and EGFR expression.

Other inhibitors that produced an effect similar to that of gedatolisib were the pan-class PI3K–AKT inhibitors pictilisib and AZD-8186, PI3Kδ-specific inhibitor idelalisib, pan-AKT inhibitor afuresertib and the mTOR1–mTOR2 inhibitor sapanisertib^31^ (Supplementary Fig. 5e). The potency of each of these PI3K–AKT–mTOR inhibitors in activating CAV1 and EGFR signaling is likely related to their target specificity and regulation in HGSOC models.

Given the validated association between CAV1 and EGFR protein expression in our HGSOC models, we researched the degree of this correlation in The Cancer Genome Atlas (TCGA) RNA-Seq dataset (*n* = 429 samples) deconvoluted for cell type (epithelial OC and fibroblasts) and International Federation of Gynecology and Obstetrics (FIGO) stages (Methods). Surprisingly, a strong *CAV1* versus *EGFR* mRNA correlation was observed in adjacent stromal cells for all disease stages, whereas, for tumor and adjacent epithelial cancer cells, the correlation was strong at the earlier disease stages only and diminished as stage progressed (Fig. 5d). Although fibroblasts exhibited higher *CAV1* expression compared to epithelial cancer cells, the proportion of cancer cells substantially outweighed that of fibroblasts (Supplementary Fig. 5f,g).

### PI3K–AKT–mTOR inhibitors improve EGFR inhibition efficacy

**Fig. 6:**
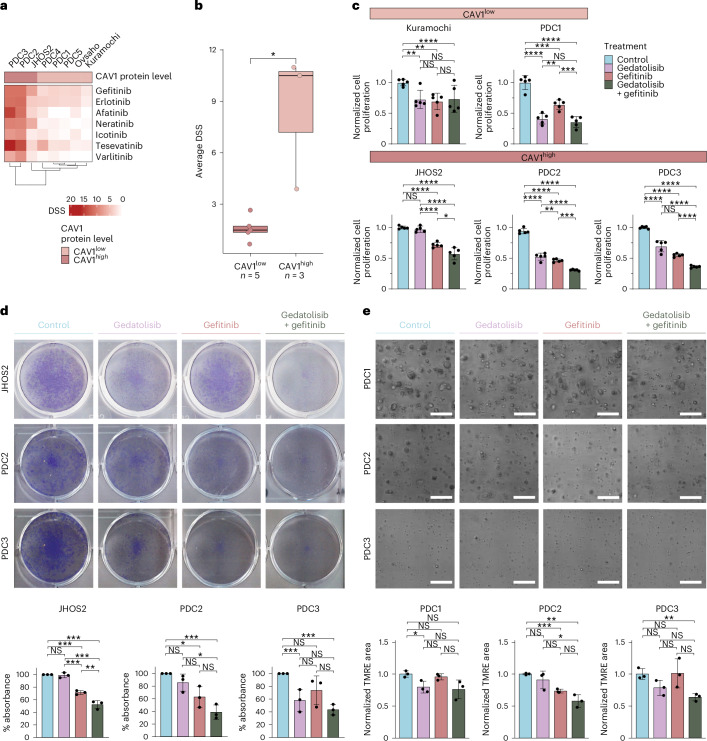
Treatment with PI3K–AKT–mTOR inhibitors augmented EGFR inhibitor cytotoxicity in CAV1^high^ HGSOC samples. **a**, Heat map of DSRT-based DSSs for seven EGFR inhibitors in eight preclinical HGSOC models. Color bars indicate the classification of CAV1 protein levels verified by immunoblotting, as in Fig. 5a. The heat map is clustered using complete-linkage hierarchical clustering based on Euclidean distances. **b**, Box plots representing the average DSS distributions of seven EGFR inhibitors for the eight models, divided according to their protein levels of CAV1. The significance of the pairwise differences between the two groups was computed using the unpaired, two-tailed Wilcoxon rank-sum test (*P < 0.05). The box plots illustrate (from top to bottom) the five-number summary of each data group: minimum average DSS, first (lower) quartile, median, third (upper) quartile and maximum average DSS. **c**, Bar charts depicting the normalized cell survival of untreated (control) or drug-treated (72 h) HGSOC cells as indicated. Results are represented as the mean ± s.d. of *n* = 5 technical replicates. For the untreated samples, the average of the detected levels was set to 1 for comparison with the treated samples. Gedatolisib concentration, 100 nM. Gefitinib concentration, 10 µM for cell lines and 5 µM for PDCs. The same concentrations were adopted for the combination treatment. **d**, Representative images of the colony-forming assay of untreated (control) or drug-treated (6 days) CAV1^high^ HGSOC models as indicated. The bar charts report the percentage absorbance at 620 nm as the mean ± s.d. of *n* = 3 technical replicates. The detected absorbance was set to 100% for the untreated (control) samples. Gedatolisib concentration, 100 nM for JHOS2, 50 nM for PDC2 and 25 nM for PDC3. Gefitinib concentration, 10 µM for JHOS2 and 500 nM for PDCs. **e**, Representative images of small-scale drug testing of PDOs. The bar charts report the normalized TMRE area as the mean ± s.d. of *n* = 3 technical replicates. The detected TMRE area was set to 1 for the untreated (control) samples. Scale bar, 100 µm. In **c**–**e**, the significance of the pairwise differences between different experimental conditions was computed using the unpaired, two-tailed Student’s *t*-test (not significant (NS), *P* > 0.05; *P < 0.05, **P < 0.01, ***P < 0.001 and ****P < 0.0001). Source data

Given the data supporting the transcriptional coregulation of *EGFR* and *CAV1* genes and interaction of corresponding proteins, we asked whether preclinical models with CAV1^high^ would report a higher sensitivity for EGFR inhibitors. Indeed, our DSRT screen uncovered a statistically significant difference between the average DSS of CAV1^high^ JHOS2, PDC2 and PDC3 compared to CAV1^low^ Kuramochi, Ovsaho, PDC1, PDC4 and PDC5 samples, as defined from their immunoblotting signal (*P* = 0.036, Wilcoxon rank-sum test; Fig. 6a,b). Thus, we investigated whether upregulation of EGFR mediated by PI3K–AKT–mTOR inhibitors would be pharmacologically useful for cotargeting approaches with EGFR-specific inhibitors such as gefitinib (IRESSA), which has been tested in multiple clinical trials in OC, either alone or in combinatorial regimens^40–42^. Treatment of HGSOC cells with gedatolisib alone elicited a variable cytotoxic effect; however, when combined with gefitinib, we observed enhanced cytotoxicity in cells with CAV1^high^ and EGFR protein expression compared to a single treatment alone (Fig. 6c). To verify whether CAV1 expression can modulate the cytotoxic effect of gedatolisib and/or gefitinib, JHOS2 and Kuramochi cells were transfected with a control or CAV1-Flag plasmid followed by drug treatments (Supplementary Fig. 6a). Our results showed that overexpression of *CAV1* in JHOS2 augmented the efficacy of gedatolisib and gefitinib and this effect was more pronounced in combinatorial gedatolisib + gefitinib sample compared to either treatment alone. However, in Kuramochi cells, the effect of *CAV1* expression only moderately impacted the effect of gedatolisib, while having no effect on gefitinib efficacy; more importantly, the augmented effect of combinatorial drug treatment compared to either treatment alone was also not observed. Furthermore, siRNA-mediated knockdown of *CAV1* expression (siCAV1) in JHOS2 cells interfered with the efficacy of combinatorial drug treatment, resulting in lower cytotoxic effect than in siRNA control-treated cells, and this effect was not observed in Kuramochi cells (Supplementary Fig. 6b). Moreover, siRNA-mediated *EGFR* knockdown in JHOS2 had a similar effect to siCAV1 on the combinatorial gedatolisib + gefitinib treatment in JHOS2 cells (Supplementary Fig. 6b).

When we applied the same treatment strategy using other PI3K–AKT–mTOR inhibitors such as afuresertib, AZD-8055, pictilisib, sapanisertib and idelalisib in combination with gefitinib, we obtained the same results, especially for PDC2 and PDC3 (Supplementary Fig. 6c). For gedatolisib in combination with gefitinib, this additive effect was also verified by long-term colony-forming assays for HGSOC models expressing CAV1 and EGFR (Fig. 6d), as well as in long-term primary patient-derived organoid (PDO) cultures using early-passage tumor samples (Fig. 6e). Thus, our results confirmed that upregulation of *CAV1* and *EGFR* mediated by PI3K–AKT–mTOR inhibitors creates a pharmacological window for cotargeting treatments in HGSOC.

## Discussion

Here, we demonstrated the utility of multiplexed scRNA-Seq using Cell Hashing^12^ for dissecting the heterogeneity of drug responses occurring in HGSOC cells. The strategy of combinatorial barcoding of live cells enables transcriptomic analyses across broad panels of drugs and samples, greatly leveraging the costs of library preparations and sequencing. This approach could be particularly useful when studying heterogeneous samples such as primary tumor samples and for uncovering mechanisms of drug resistance. Ex vivo methodologies using PDCs as ‘living biobanks’ produce data of notable clinical relevance and, when combined with single-cell pharmacotranscriptomics profiling, the translational importance increases manifold. Thus, these approaches can identify possible treatment strategies for patients even in the absence of a complete understanding of the molecular underpinnings^43^.

Our pipeline for pharmacotranscriptomics profiling combined DSRT screens of HGSOC cells with 96-plex scRNA-Seq across 288 samples covering 45 drugs with 13 distinct MOAs, chosen for their targeting of pathways commonly altered in HGSOC. Specifically, our aim was to validate the feasibility of our approach by investigating the origin and extent of the transcriptional changes elicited by distinct MOAs and whether drug response phenotypes induced in HGSOC samples differ among samples collected from different sites (from ovary tissue or ascites) and from patients at different treatment phases (from post-NACT or relapsed cases).

We observed different scales of DGE, ranging from larger perturbations induced by drugs such as CDK, HDAC and BET inhibitors to smaller differences induced by PARP inhibitors and SMAC mimetics. Although the transcriptional dynamics as measured by DGE in response to drug treatment may not directly correlate with cytotoxic efficacy as previously demonstrated^27^, we could derive insights into drug MOAs at the mRNA level and possible adaptive or resistance mechanisms unanticipated from bulk cell viability readouts alone. For instance, our analyses showed that for drugs targeting intracellular signaling (for example, PI3K–AKT–mTOR and BCL-2 inhibitors), a model-specific drug response phenotype is prevalent, whereas epigenetics modifiers *(*for example, BET and HDAC inhibitors) elicit a shared drug response among cell lines and PDCs. On the other hand, a common downregulation of HR pathways was observed for all MOAs, which could be related to the HGSOC genomic architecture harboring broader HR defects in almost 50% of cases^44^.

The PI3K–AKT–mTOR pathway is frequently and aberrantly activated in many cancers, including OC; thus, it represents an attractive target for therapeutic intervention^31^. Despite the growing number of preclinical studies and early-phase trials, targeting various effectors of PI3K–AKT–mTOR signaling has not resulted in a treatment update in OC^31,45^. Resistance to PI3K–AKT–mTOR inhibitors is often mediated by feedback loops such as (1) rewiring of the existing signaling pathways to neutralize or balance the inhibitory effects through compensatory upregulation of RTKs^46,47^; (2) FOXO-regulated transcription and cap-independent translation mediating the upregulation of cellular survival effectors such as *BCL2*, *EGFR* or *IGF1R* (refs. ^48,49^); and (3) the activation of insulin signaling following relief of the negative feedback loop from *SGK*1 to *IRS1* (ref. ^50^). Our transcriptomic analyses identified multiple effectors of these resistance mechanisms such as modulation of *MET*, *IRS1*, *IRS2*, *INSR1*, *FOXO3* and *EGFR* by PI3K–AKT–mTOR inhibitors, indicating that multiple response mechanisms are occurring at the same time, as expected.

Modulation of *CAV1* expression by PI3K–AKT–mTOR inhibitors was the most intriguing finding in our analyses. CAVs and cavins are two families of proteins constituting the structural components of caveolae (that is, cavities of the cell membrane participating in a variety of biological processes such as signal transduction and mechanical stress response), providing a signaling platform for tumor cell growth and resistance to apoptotic stimuli^51,52^. In particular, CAV1 is a multifactorial scaffolding protein able to remodel the extracellular environment and is known to be involved in tumor progression^53^. How PI3K–AKT–mTOR inhibitors could induce *CAV1* and *EGFR* expression in HGSOC cells is not fully understood. We postulate that a transcriptional feedback loop could mediate *CAV1* induction, as public chromatin immunoprecipitation and sequencing (ChIP-Seq) data identified several transcription factors binding sites at the *CAV1* promoter (*EGR1*, *MYC*, *MYCN* and *TP53*; Supplementary Fig. 6d). In turn, *CAV1* upregulation induces *EGFR* expression, as previously shown in multiple cancers^36,38^ and demonstrated by our data. However, *EGFR* expression is also required for this feedback loop to work, as targeted knockdown of *EGFR* appears to impair gedatolisib-mediated *CAV1* upregulation. Because previous studies have shown that EGFR interacts with CAV1 at the plasma membrane, a mechanism that involves Ras–Raf intracellular signaling and EGFR trafficking^54^, it is plausible that PI3K–AKT–mTOR inhibitors induce a feedback loop through *CAV1* and *EGFR* upregulation that is dependent on the CAV1–EGFR interaction in cells. Several CAV1 isoforms have been previously identified and we confirmed that all the main isoforms are expressed in our HGSOC ex vivo models, with the longer CAV1 isoform (178 aa) being the most abundant (Supplementary Fig. 6e).

Our findings could have translational importance in HGSOC from two perspectives. Firstly, CAV1 and EGFR protein expression could be used as a marker for the efficacy of EGFR inhibitors. Secondly, upregulation of *CAV1* and *EGFR* expression mediated by PI3K–AKT–mTOR inhibitors augments the effectiveness of EGFR inhibitors in HGSOC to elicit an additive effect with enhanced cytotoxicity. A phase 2 clinical study of the EGFR inhibitor gefitinib showed decreased pAKT and pERK levels in samples from persons with OC; however, the clinical effects were modest, likely because of a lack of patient selection^42^. Nevertheless, the study suggested that, while gefitinib as a monotherapy has limited clinical activity, combinatorial treatment with molecular therapeutics against complementary targets may prove successful.

In conclusion, our 96-plex drug testing coupled with scRNA-Seq provided important insights stemming from pharmacotranscriptomic analyses of drug responses in HGSOC models, including ex vivo samples. Our experimental validations of the responses to PI3K–AKT–mTOR inferred from scRNA-Seq analysis suggest a key role for *CAV1* and *EGFR* expression in modulating a regulatory feedback loop in HGSOC drug resistance. In turn, the identification of multiple additive effects of PI3K–AKT–mTOR inhibitors and gefitinib for HGSOC with *CAV1* and *EGFR* expression offers a better approach to tailor new combinatorial treatments in HGSOC.

## Methods

### Cell lines and PDC cultures

The cell line JHOS2 (Riken BioResource Research Center) was grown in DMEM/F12 (21331020, Gibco, Thermo Fisher Scientific) supplemented with 10% FBS (11573397, Gibco, Thermo Fisher Scientific), 1% UltraGlutamine (BE17-605E/U1, Lonza), 1% MEM nonessential amino acids solution (11140050, Gibco, Thermo Fisher Scientific) and 0.01% Primocin (ant-pm-2, Invivogen). OC cell lines Kuramochi and Ovsaho were obtained from the Japanese Collection of Research Bioresources and grown in RPMI-1640 medium (31870025, Gibco, Thermo Fisher Scientific) supplemented with 10% FBS, 1% UltraGlutamine and 0.01% Primocin. Information on the authentication of cell lines is available in the Nature Portfolio Reporting Summary and their cancer drivers can be found at https://cellmodelpassports.sanger.ac.uk/. The patient material and data were acquired upon informed consent and under institutional ethical review board-approved protocols (no. 56/13/03/03/2014) at the University of Helsinki Central Hospital. The PDCs were cultured as previously described^25^ but without feeder cells, either in DMEM/F12 (31330038, Gibco, Thermo Fisher Scientific) supplemented with 1× B27 (17504044, Gibco, Thermo Fisher Scientific), 20 ng ml^−1^ EGF (354052, Corning), 10 ng ml^−1^ fibroblast growth factor (PHG0023, Gibco, Thermo Fisher Scientific) and 0.02% Primocin or in 75% F12 (21765029, Gibco, Thermo Fisher Scientific) and 25% DMEM (41965039, Gibco, Thermo Fisher Scientific) supplemented with 5% FBS, 0.4 µg L^−1^ hydrocortisone (H0888, Sigma-Aldrich), 5 µg ml^−1^ insulin (I0516, Sigma-Aldrich), 8.4 ng ml^−1^ cholera toxin (C8052, Sigma-Aldrich), 10 ng ml^−1^ EGF, 24 µg ml^−1^ adenine (A2786, Sigma-Aldrich), 0.02% Primocin and 10 µM Y-27632 (ALX-270-333, Enzo Life Science). PDCs were grown on Primaria plates (353846, Corning). Cell counting was performed using a TC20 automated cell counter (1450102, Bio-Rad Labotories). During drug treatment, Y-27632 was not used. The PDCs were verified for identical phenocopy with their original tumor samples by next-generation sequencing (data available upon request) and the immunohistochemistry-based expression of the Müllerian marker *PAX8*, a known marker for the diagnosis of epithelial OCs. All HGSOC PDCs displayed a loss-of-function *TP53* mutation and PI3K pathway aberration (*PI3KCA* gain or amplification).

### Cell Hashing and scRNA-Seq

#### Sample preparation and Cell Hashing labeling

The HGSOC cells from JHOS2 and the two PDCs (PDC2 and PDC3) were harvested according to standard cell culture procedures. After medium removal, the cells were washed with 6 ml of PBS and detached with 1 ml of TrypLE express enzyme (12605010, Gibco, Thermo Fisher Scientific) incubation for 10–15 min at 37 °C and 5.0% CO_2_ before quenching with 5 ml of warm medium. During incubation, the preprinted drugs in the 96-well plates were dissolved in 25 µl of medium while shaking for 15 min. The cell suspension was then transferred to a 15-ml tube and centrifuged at 300*g* for 5 min. After removing the supernatant, the pellet was resuspended in 5 ml of warm medium before cell counting, which was performed as described before. The objective was to plate ~13,000 cells per well by adding 75 µl of the cell suspension to each well after dissolving the drugs to obtain a final volume of 100 µl per well of each 96-well plate. Finally, cells were incubated for 24 h at 37 °C and 5.0% CO_2_ before harvesting for Cell Hashing^12^. Live-cell labeling was performed as previously described^12,55^ with pairwise combinations of 20 in-house-produced antibody–oligo conjugates targeting B2M (316302, BioLegend) and CD298 (341712, BioLegend). The full list of drugs used for multiplexed scRNA-Seq, along with their concentrations, identifiers, sources and solvents, is reported in Supplementary Table 1 and the sequences of the 90-nt DNA barcodes conjugated to the antibodies are available in Supplementary Table 3.

#### Library chemistry, sequencing and raw FASTQ file preprocessing

Single-cell gene expression profiles were studied using the 10x Genomics Chromium single-cell 3′ RNA-Seq platform (10x Genomics). The Chromium single-cell 3′ RNA-Seq run and library preparation were performed using Chromium Next GEM single-cell 3′ gene expression version 3.1 dual-index chemistry with feature barcoding technology (CG000317, 10x Genomics). The sample libraries were sequenced on the Illumina NovaSeq 6000 system (Illumina) using read lengths of 28 bp (read 1), 10 bp (i7 index), 10 bp (i5 index) and 90 bp (read 2). The target minimum coverage was 50,000 reads per cell. Raw FASTQ file preprocessing was performed using Cell Ranger (version 7.1.0, 10x Genomics) pipelines. Specifically, cellranger mkfastq was used to produce FASTQ (raw sequence data) files and cellranger count was used to perform alignment, filtering and unique molecular identifier (UMI) counting. Alignment was performed against the GRCh38 (GENCODE version 32, Ensembl 98)^56^ assembly of the human genome.

### scRNA-Seq data demultiplexing and analysis

The scRNA-Seq data bioinformatic analyses were performed using R (4.2.2)^57^, primarily using the R package Seurat (4.3.0)^58^. First, only cells for which both HTO and mRNA data were available were retained. Then, the HTO data were normalized for each of the three batches individually using the function ‘NormalizeData’ with the centered log-ratio transformation. Then, cells were demultiplexed using the function ‘HTODemux’ with default parameters for row-specific and column-specific HTOs, separately. Only cells classified as ‘singlets’ for both the row-specific and the column-specific HTOs were retained, whereas all the negative and doublet cells were discarded. A breakdown of the total number of cells before and after the demultiplexing can be found in Supplementary Table 4. The remaining pool of cells from the HTO classification was subjected to quality controls according to the number of UMIs (nCount_RNA > 2,500 and nCount_RNA < 80,000) and percentage of UMIs from mitochondrial genes (percent.mt < 25 for JHOS2 percent.mt < 30 for PDC2 and percent.mt < 20 for PDC3). After quality control filtering, the batch data were merged for downstream preprocessing. Normalization and variance stabilization of the three batches of scRNA-Seq data were performed using SCTransform v2 (ref. ^59^), regressing out the percentage of counts accounting for ribosomal and mitochondrial protein-coding genes, as well as the number of genes and UMIs. Cell-cycle heterogeneity was scored after a first round of variance stabilization and normalization with ‘CellCycleScore’, using the cell-cycle phase references provided by Tirosh et al.^60^ Subsequently, the data were renormalized and stabilized by regressing the cell-cycle scoring. Principal component analysis followed on the single-cell transcriptomic data and the batch effect was corrected with the R package Harmony (version 0.1.1)^61^. After constructing the shared nearest neighbor network with ‘FindNeighbors’ on the Harmony batch-corrected dimensionality reduction (reduction = ‘harmony’), clustering was performed through use of the Leiden algorithm^62^ with all the possible resolutions between 0 and 1 and a step size of 0.1; then, ‘clustree’, from the clustree R package (version 0.5.0)^63^, was used to choose the best resolution (0.3). UMAP embeddings were computed using the ‘RunUMAP’ Seurat function with reduction = ‘harmony’. For all the UMAPs generated, min.dist was set to 0.5, dims was set to 1:30 and the number of neighbors was set to 5. When subsetting for model-specific cells, the data were renormalized and variance-stabilized, without correcting for the batch effect.

Differential expression analyses for the identification of Leiden cluster markers were run using ‘FindAllMarkers’ and adopting the Wilcoxon rank-sum test (test.use = ‘wilcox’). *P* values were adjusted with Bonferroni correction using all the features in the dataset as default. GSVA^64^ was conducted using the ‘gsva’ function from the R package oppar (0.99.8)^65^ against the MSigDB GO:BP C5 collection, as downloaded on March 24, 2023. Gene sets from the C5 collection were filtered to those with at least 10 genes and at most 500 genes, resulting in 834 final gene sets from a total of 7,751. GSVA was calculated using the average expression of the 50 most upregulated and downregulated genes for each of the 13 Leiden clusters as previously computed with ‘FindAllMarkers’. The significance of the scores was calculated by bootstrapping 1,000 times. The top 5% most variable terms were selected by calculating the coefficient of variation of each gene set retained in the analysis. The 3D UMAPs were produced using the R package plotly (version 4.1.10)^66^ as described by Qadir et al.^67^

### DGE analysis using subsample pseudobulk aggregation

Each model and the 46 treatment groups were divided into subsets or subsamples of ten cells^68^. If the number of cells in a group wasn’t divisible by ten, only subsets with at least five cells were kept. It was ensured that each treatment group had at least two subsets. Consequently, treatment groups with 14 or fewer cells would have been excluded but none were, as even the smallest group had 29 cells. Subsequently, we calculated the pseudobulk expression of each subsample of cells using ‘AggregateExpression’ from Seurat on raw counts. For each of the three models, differential expression analyses were performed using the R packages edgeR (version 3.40.2)^69^ using the genewise negative binomial generalized linear models with quasilikelihood tests with the functions ‘glmQLFit’ and ‘glmQLFitTest’. Specifically, after filtering out lowly expressed genes (average log_2_(counts per million) < 1 in the dataset containing the compared subsamples), normalization factors were calculated with ‘calcNormFactors’ and a design matrix was prepared to model the experimental groups. Negative binomial dispersions were estimated with ‘estimateDisp’ and a quasilikelihood negative binomial generalized linear model was fitted to the data. A quasilikelihood *F*-test with ‘glmQLFitTest’ was performed to compare treatment groups against controls and the genes were extracted with adjusted *P* values using the Benjamini–Hochberg method. ORA was performed using gprofiler2 (version 0.2.1)^70^; protein-coding genes that were found significantly upregulated or downregulated (|log_2_FC| > 0.25, FDR < 0.01) were taken from the DGE analyses results. For these comparisons, the pseudobulk aggregates approach outperformed the canonical one adopted for scRNA-Seq data comparisons, such as the Wilcoxon rank-sum test, in detecting the upregulation of protein-coding genes (Supplementary Fig 3c). Here, for each MOA, the union of the upregulated protein-coding genes across all drugs with an MOA was taken and the same was applied for the downregulated genes. Finally, the two gene sets were independently submitted to ORA against the Reactome^28^ database, with *Homo sapiens* as the organism and an FDR threshold of 0.05. The results were filtered for a custom list of cancer-related Reactome pathways derived by merging the pathways identified in the Reactome cancer entry (database identifier: 1500689), pathways significantly enriched in the evolutionary states from Lahtinen et al.^30^ and Reactome pathways containing the terms ‘FOXO’ or ‘insulin’ (to include terms related to mechanisms of resistance present in HGSOC; Supplementary Table 2). Lastly, if more than 20 Reactome pathways were retained for upregulated or downregulated genes, only the 20 most significant pathways were retained for each of the two sets; for repeated terms, the first and most significant instance was retained.

### LINCS perturbagen data analyses

A total of 1,436,310 experiment results from the LINCS L1000 chemical perturbations characteristic up and down gene sets were retrieved from the SigCom database^71^. In these characteristic sets, the top 250 upregulated and downregulated genes for each experiment are presented with experimental conditions. The perturbation experiments were filtered to those that (1) involved a perturbagen classified with an MOA defined as either an ‘AKT inhibitor’ or ‘PI3K inhibitor’; (2) were performed in a cell line from ovary or breast; and (3) had a time point of 24, 48, 72, 96 or 120 h. As a result, 2,254 experiments satisfied these conditions. Count matrices were calculated for all genes that occurred in the top upregulated or downregulated gene sets among the target 2,254 experiments to identify the most commonly perturbed genes by PI3K–AKT inhibition in ovary and breast cells over 24–120 h. Characteristic gene sets for RTK signaling and PI3K–AKT signaling pathways were retrieved from MSigDB^72^, as part of the C2 curated gene sets, labeled as ‘Reactome signaling by RTKs’ and ‘WP PI3K–AKT signaling pathway’, respectively.

### Immunoblotting and immunoprecipitation

For the drug treatment experiments, in the case of the representative cell lines, 250,000 cells per well were seeded to a six-well plate and the cells were treated for 48 h with 100 nM and 500 nM gedatolisib (s2628, Selleck Chemicals), whereas PDCs were treated with 50 nM and 100 nM gedatolisib. An equivalent volume of PBS was added to control (untreated) wells. Cells were lysed with Triton X-based lysis buffer (50 mM Tris-HCl pH 7.5, 10% glycerol, 150 mM NaCl, 1 mM EDTA, 1% Triton X-100 (X100-100ML, Sigma-Aldrich) and 50 mM NaF) supplemented with protease and phosphatase inhibitors (phosphatase inhibitor cocktail (two tubes, 100×; B15001) and protease inhibitor cocktail (EDTA-free, 100× in DMSO; B14001), both from Bimake (now Selleck Chemicals)). For immunoprecipitation, cell lysates were incubated overnight at 4 °C with EGFR (4267, Cell Signaling Technology (CST); 1:100) and 1 µg of Ultra-LEAF purified recombinant human IgG1 isotype control (403501, BioLegend) was used as a control. Then, the samples were incubated for 1 h at 4 °C with prewashed protein A agarose beads (9863, CST; 1:4), after which the beads were mixed with 2× Laemmli sample buffer (1610737, Bio-Rad Laboratories) and separated by SDS–PAGE followed by immunoblotting. Briefly, separated proteins from both immunoblotting and immunoprecipitation were transferred to 0.45-µm nitrocellulose membranes and the following primary antibodies were used (all 1:1,000 dilution): anti-β-tubulin (86298, CST), anti-AKT (2920, CST), anti-pAKT (S473) (4060, CST), anti-CAV1 (3267, CST), anti-EGFR (4267, CST), anti-ERK1–ERK2 (4696, CST) and anti-pERK1–pERK2 (9101, CST). Secondary antibodies were IRDye 800CW donkey anti-mouse IgG and IRDye 680RD donkey anti-rabbit IgG (926-32212 and 926-68073, LI-COR) or AzureSpectra fluorescent secondary antibodies goat anti-mouse 550 (AC2159, Azure Biosystems). The membranes were scanned with the Odyssey Fc imaging system (LI-COR) or Azure 600 (Azure Biosystems). Image analysis was performed using Image Studio Lite (LI-COR).

### Immunofluorescence staining

A total of 1,000 JHOS2 and 5,000 Kuramochi cells were seeded on 12-mm glass coverslips on 24-well plates for 24 h. In the case of PDCs, 30,000 PDC1, PDC2 and PDC3 cells were seeded. Cells were treated with 500 nM afuresertib (FIMM136448, FIMM), AZD-8186 (A-1610, Active Biochem), gedatolisib, idelalisib (FIMM003750, FIMM), pictilisib (HY-50094, MedChemExpress) and sapanisertib (FIMM109442, FIMM) for 48 h. Then, the cells were fixed for 10 min with 4% paraformaldehyde pH 7.4 (158127, Sigma-Aldrich) followed by permeabilizing the cells with 0.1% Triton X-100 solution in PBS for 10 min. After blocking the cells with 1% BSA (P6154, BioWest) for 1 h at room temperature, cells were incubated overnight with anti-EGFR and anti-CAV1 antibodies (as used for immunoblotting; 1:500) at 4 °C. As a secondary antibody, donkey anti-rabbit IgG (H + L) highly cross-adsorbed secondary antibody, AlexaFluor Plus 594 (A32754, Thermo Fisher Scientific; 1:1,000) was used, whereas DAPI (D9542, Sigma-Aldrich) was used for nuclear staining. The glass coverslips in the 24-well plate were picked and mounted on glass slides with Immu-Mount (9990402, Fisher Scientific). Images were captured with LSM700 Confocal Microscope (Carl Zeiss) and inspected with ZEN Blue digital imaging for light microscopy (version 3.5.093, Carl Zeiss).

### DSRT and small-scale drug combination testing

DSRT and the calculation of the DSSs were performed as described previously^26,73^. In short, 1,000–1,500 cells were added to wells of 384-well plates with drugs preplated with five concentrations over a 10,000-fold concentration range. After a 3-day incubation at 37 °C and 5.0% CO_2_, cell viability was measured using the CellTiter-Glo 2.0 assay (G9242, Promega), luminescence was read with a PHERAstar FS (BMG Labtech) and the outcoming data were analyzed using Breeze^74^ (https://breeze.fimm.fi/). For small-scale drug combination testing, 1,200 cells were seeded to a 384-well plate in 25 µl of medium, including the drugs or drug combinations in desired concentrations. Gefitinib was used at a concentration of 1 µM for PDCs and 10 µM for cell lines, while other drug (gedatolisib, afuresertib, AZD-8186, idelalisib, pictilisib and sapanisertib) concentrations varied depending on their properties; concentrations are reported in the relative figure legends. Five technical replicates were used for each sample. After 72 h of incubation, cell viability was measured as described above with CellTiter-Glo 2.0 and luminescence was read with PHERAstar FS or Spark multimode microplate reader (Tecan Group). The CellTiter-Glo data analysis was conducted using GraphPad Prism software (version 9.3.1) to obtain comprehensive results and statistical analysis. The s.d. was chosen over the s.e.m. to provide a measure of the variability within the data points rather than the precision of the estimated mean.

### Image-based small-scale drug testing of PDOs

Early-passage PDCs were embedded in 25 µl of 66% VitroGel ORGANOID-4 (VHM04-4, TheWell Bioscience) according to the manufacturer’s recommendations on a 384-well plate (ultralow attachment, Corning), with 5,000 cells per well. The plate was incubated at 37 °C for 15 min followed by the addition of 25 µl of top medium. The top medium was refreshed every 4 days. Organoids were allowed to form over 14 days before treatment using the following drugs: 50 nM gedatolisib and 500 nM gefitinib as monotreatment or in combination, diluted in medium. As a control, an equivalent volume of DMSO was added and three replicates were used. After 12 days of drug exposure, cells were live-stained with 75 nM TMRE (Abcam), and *z*-stacked images were taken using Leica THUNDER Imager live cell and 3D assay (Leica). The images were analyzed using CellProfiler (version 4.2.1)^75^ and the standard pipeline (including modules ‘IdentifyPrimaryObjects’ and ‘MeasureImageAreaOccupied’) was used to analyze the area of the image covered with the TMRE signal to quantify the viability of organoids in each condition. Results were standardized to the control.

### siRNA-mediated silencing and overexpression of *CAV1* and *EGFR*

Transient silencing of *CAV1* and/or *EGFR* was achieved by plating JHOS2 and Kuramochi cells in six-well plates with 200,000 cells per well for immunoblotting analyses and in 96-well plates with 8,000 cells per well for cell viability assays. In both settings, the cells were allowed to adhere to the plates for 24 h, after which the transfections were performed. For the silencing of *CAV1* (L-003467-00-0005, Dharmacon Reagents), *EGFR* (L-003114-00-0005, Dharmacon Reagents) or the control (D-001810-10-05, Dharmacon Reagents), an ON-TARGET^plus^ siRNA smart pool of four siRNAs per target was used at the final concentration of 12.5 nM using DharmaFECT 1 transfection reagent (T-2001-02, Dharmacon Reagents). For plasmid transfections, *CAV1* was cloned with a Flag tag into the pCDNA3.1 expression plasmid and 2 µg of purified DNA per well in six-well plates or 100 ng of purified DNA per well in 96-well plates was transfected using FuGENE HD transfection agent (E2311, Promega) as per the manufacturer’s instructions. Equal amounts of pSG5 plasmid (216201, Agilent Technologies) were used in transfections as a control. Drug treatment was performed 24 h after transfections at the concentrations indicated in the figure legends. Then, 48 h after the drug introduction, the cells were harvested by whole-cell lysis and used for immunoblotting or subjected to cell viability assay as described above.

### Colony-forming assay

Depending on the growth rate of the cell model, 5,000–20,000 cells per well were plated in 12-well plates. Then, 24 h later, PDCs were treated with either gedatolisib (PDC2, 50 nM; PDC3, 25 nM) or gefitinib (500 nM) as monotherapy or in combination; the representative cell lines were treated with gedatolisib (100 nM) and gefitinib (10 µM) alone or in combination. The growth medium and drugs were refreshed every third day. Treatment continued for 8–12 days until the untreated cells were confluent. Subsequently, the cells were fixed with a solution of methanol and acetic acid (7:1) and stained with 0.5% crystal violet (C3886, Sigma-Aldrich). The images of the plates were taken using imageRUNNER ADVANCE C2225i (Canon) for qualitative analysis followed by eluting the bound crystal violet stain with 100% methanol (10653963, Fisher Chemical). The absorbance of the elute was measured at 620 nm using the Spark multimode microplate reader (Tecan Group).

### Bulk RNA-Seq library chemistry, sequencing and raw FASTQ files preprocessing

Total RNA was isolated from the PDCs using the RNeasy kit (Qiagen). The quantity and quality of the RNA samples were assessed by Qubit (Thermo Fisher Scientific) and Bioanalyzer (Agilent Technologies). RNA with an RNA integrity number > 8 was used for subsequent analysis. Libraries were multiplexed and paired-end sequencing was performed with the Illumina HiSeq system (Illumina). Resulting FASTQ files were aligned to the GENCODE version 36 (GRCh38)^76^ assembly of the human genome with the STAR RNA-Seq aligner (version 2.7.10b)^77^. STAR run parameters were defined as follows: ‘--twopassMode Basic’, ‘--alignSJoverhangMin 8’, ‘--alignSJDBoverhangMin 1’, ‘--outFilterMismatchNoverLmax 0.1’, ‘--alignIntronMin 20’, ‘--alignIntronMax 1000000’, ‘--outFilterScoreMinOverLread 0.33’, ‘--outFilterMatchNminOverLread 0.33’, ‘--limitSjdbInsertNsj 1200000’, ‘--outSAMstrandField intronMotif’, ‘--chimSegmentMin 15’, ‘--chimJunctionOverhangMin 15’ and ‘--chimMainSegmentMultNmax 1’; all other parameters were default.

### Cell type deconvolution of OC from TCGA

Sample and clinical data and raw counts for 429 bulk RNA-Seq analyses were downloaded from TCGA OC dataset (TCGA-OV)^29,78^. Unstranded counts were extracted from all experiments and combined. scRNA-Seq count data were downloaded for 43,945 ovary cells with associated cell metadata annotations, including cell type (endothelium, epithelium, erythrocyte, fibroblast, lymphocyte, myeloid, plasma or undefined) and sample location (normal, tumor or tumor-adjacent)^79,80^. TCGA-OV bulk RNA-Seq count data were then deconvoluted using the R package BayesPrism (version 2.0)^81^ and the scRNA-Seq count and annotation data were used to identify overall cell type composition and gene expression by cell type for all 429 samples. Deconvoluted raw counts were subsequently normalized with the ‘vst’ function of the R package DESeq2 (version 1.40)^82^ and then normalized to transcripts per million (TPM) based on GENCODE version 36 gene exon annotations.

### CAV1 transcript analysis

Data for 11,045 ChIP-Seq experiments that were uniformly processed with MACS2 (ref. ^83^) were retrieved as BED files from the Gene Transcription Regulation Database^84^. ChIP-Seq peak intervals were mapped to the promoter of the *CAV1* gene (Ensembl release 109 annotation) for *MYC* (147 experiments), *MYCN* (47 experiments), *EGR1* (26 experiments) and *TP53* (159 experiments) with *CAV1* transcripts plotted using the R package genemodel (version 1.10)^85^. Transcript-specific expression values as TPM were downloaded for bulk RNA-Seq experiments cataloged in the Genotype-Tissue Expression project (version 8)^86^ to compare the expression values of *CAV1* transcripts in 195 normal ovary samples with those of PDC1, PDC2 and PDC3. Transcript-specific expression values for PDC1, PDC2 and PDC3 were quantified using the ‘quant’ function of the kallisto program (version 0.46.1)^87^ with Ensembl genomes version 96 as the index.

### Statistical information

The statistical techniques used for scRNA-Seq and RNA-Seq analyses were elucidated in the preceding sections. Pertinent details concerning the statistical analyses were incorporated within the figure legends or described in the Methods, wherever applicable.

### Reporting summary

Further information on research design is available in the Nature Portfolio Reporting Summary linked to this article.

## Online content

Any methods, additional references, Nature Portfolio reporting summaries, source data, extended data, supplementary information, acknowledgements, peer review information; details of author contributions and competing interests; and statements of data and code availability are available at 10.1038/s41589-024-01761-8.

## Supplementary information

Supplementary Information Supplementary Figs. 1–6 and unprocessed scans of Supplementary Fig. 5a–d.

Reporting Summary

Supplementary Tables 1–4 Supplementary Tables 1–4.

Supplementary Data 1 CellTiter-Glo values, *P* values, *t* values and degrees of freedom of the statistical tests used in Supplementary Fig. 6a–c.

## Source data

Source Data Fig. 1 DSS for the 45 compounds included in the library for multiplexed scRNA-seq.

Source Data Fig. 3 gProfiler ORA results for each model, MOA and upregulated and downregulated genes.

Source Data Fig. 5 Unprocessed western blots.

Source Data Fig. 6 DSS for the tested EGFR inhibitors. CellTiter-Glo, colony-forming assay and TMRE values. *P* values, *t* values and degrees of freedom of the statistical tests.

Source Data Extended Data Fig. 1 GSVA scores and bootstrapped *P* values for the 13 Leiden clusters.

## Data Availability

Raw (only for JHOS2) and processed scRNA-Seq data from this manuscript were deposited to the National Center for Biotechnology Information Genome Expression Omnibus under accession number GSE274905. The Leiden cluster markers, the subsample pseudobulk DGE analysis results, the patient-derived bulk RNA-Seq processed data and the interactive 3D UMAPs are available from Mendeley Data (10.17632/j9j4mdm9yr.1). The raw FASTQ files used to generate results for the patient-derived models will be provided for scientific research upon reasonable request to fulfill privacy and ethical concerns. Source data are provided with this paper.

## Code availability

All relevant code is available from GitHub (https://github.com/ungureanulab/pharmacotranscriptomics).
