# Supplementary material for: A multiplex single-cell RNA-Seq pharmacotranscriptomics pipeline for drug discovery
Source: Nat Chem Biol. 2024 Oct 31;21(3):432–42. doi: 10.1038/s41589-024-01761-8 (PMC11867973; doi:10.1038/s41589-024-01761-8)

nature chemical biology

Supplementary information https://doi.org/10.1038/s41589-024-01761-8

A multiplex single-cell RNA-Seq pharmacotranscriptomics pipeline for drug discovery

In the format provided by the authors and unedited

Table of Contents

Supplementary Figure 1	Background information on the study design, genomic alterations, and drug sensitivity profiles of the HGSOC samples analyzed in the study.
Supplementary Figure 2	Overview of the 96-plexed scRNA-Seq output.
Supplementary Figure 3	Subsample pseudobulk aggregates to mitigate small sample sizes in differential gene expression analysis.
Supplementary Figure 4	PI3K/AKT/mTOR inhibitors modulate the transcriptional regulation of caveolae-related genes and RTKs found in their genomic vicinity.
Supplementary Figure 5	CAV1 and EGFR are modulated by PI3K/AKT/mTOR inhibitors in CAV1/EGFR expressing HGSOC.
Supplementary Figure 6	Combinatorial therapy using PI3K/AKT/mTOR inhibitors and gefitinib shows higher cytotoxicity than monotherapy in CAV1/EGFR expressing HGSOC cells.
Whole Western blots scans	Unprocessed scans of Supplementary Figures 5a–d.

Supplementary Fig. 1

a

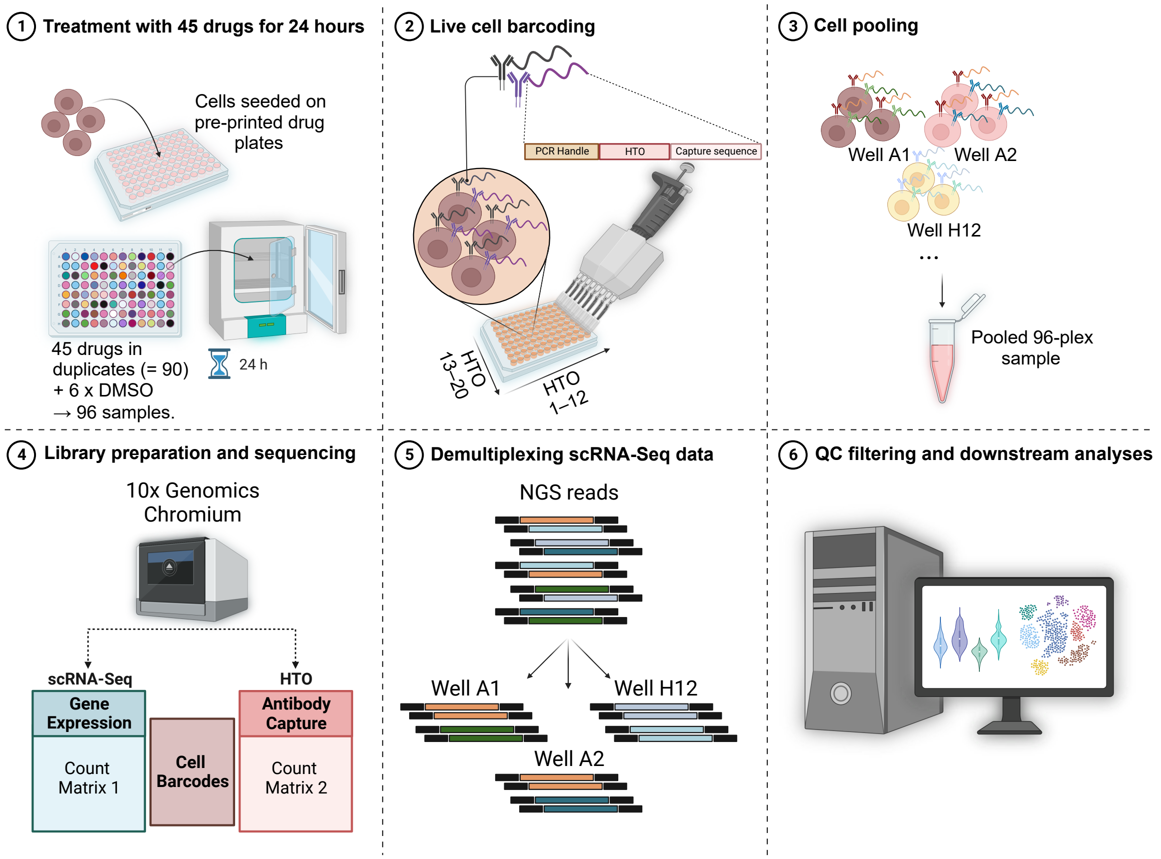

b

Sample	*FIGO stage*	*Sample type*	*Cancer drivers*	*Treatment phase*
PDC1	IVA	Ascites	TP53 p.R175H, CCNE1 Amp, MECOM Amp, PIK3CA gain	Relapsed
PDC2	IVA	Tissue	TP53 frameshift, MYC Amp, KRAS Amp, PIK3CA gain, ING5-THAP4 fusion, ROCK1-SS18 fusion	Post-NACT
PDC3	IIIC	Tissue	TP53 p.R283P, CCNE1 Amp, MECOM Amp, PIK3CA Amp, FNBP4-PTPMT1 fusion	Post-NACT
PDC4	IIIC	Ascites	TP53 p.A161T, broad CNV changes incl. PIK3CA gain	Relapsed
PDC5	IIIC	Ascites	TP53 p.R273C, CCNE1 and KRAS Amp, PIK3CA gain, RCC1-UBE2D2 fusion	Relapsed

c

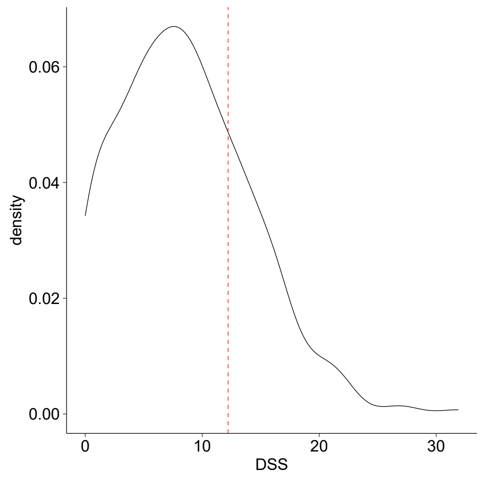

Supplementary Fig. 1: Background information on the study design, genomic alterations, and drug sensitivity profiles of the HGSOC samples analyzed in the study. a To investigate the transcriptional landscape withstanding drug treatment in HGSOC, we performed high-throughput pharmacotranscriptomic profiling using multiplexed single-cell RNA-sequencing (scRNA-Seq) after 24 hours of drug treatment. Using pairwise combinations of 20 antibody-oligonucleotide conjugates against the ubiquitously expressed, cell membrane proteins CD298 and $\beta_2$microglobulin (B2M), the live content of each well of a 96-well plate was uniquely labelled. Subsequently, cells could be pooled and submitted to library preparation and sequencing. The count data coming from the number of reads aligning to the barcodes is used to demultiplex the data, prior to the state-of-the art scRNA-Seq filtering and data analyses. **b** Table reporting the FIGO stage, sample type, key oncogenic aberrations, and treatment phase of the 5 PDCs used in this study, as derived from whole exome sequencing and copy-number variant analyses. **c** Density distribution of the DSSs of the 45 drugs and 8 pre-clinical models (5 PDCs and 3 representative cell lines) included in DSRT (*n* = 360). To aid interpretation, a dashed line is plotted at x = 12.2, representing the 75th percentile of the DSS distribution. This threshold denotes the point of DSS value at which drugs begin to be recognized as exhibiting a heightened sensitivity response.

Supplementary Fig. 2

a

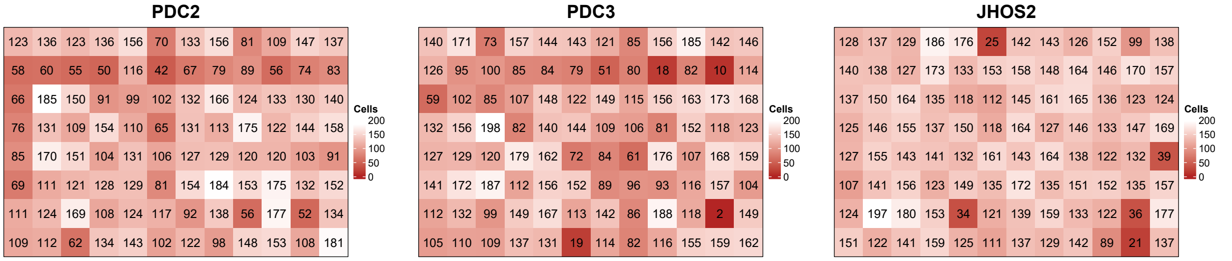

b

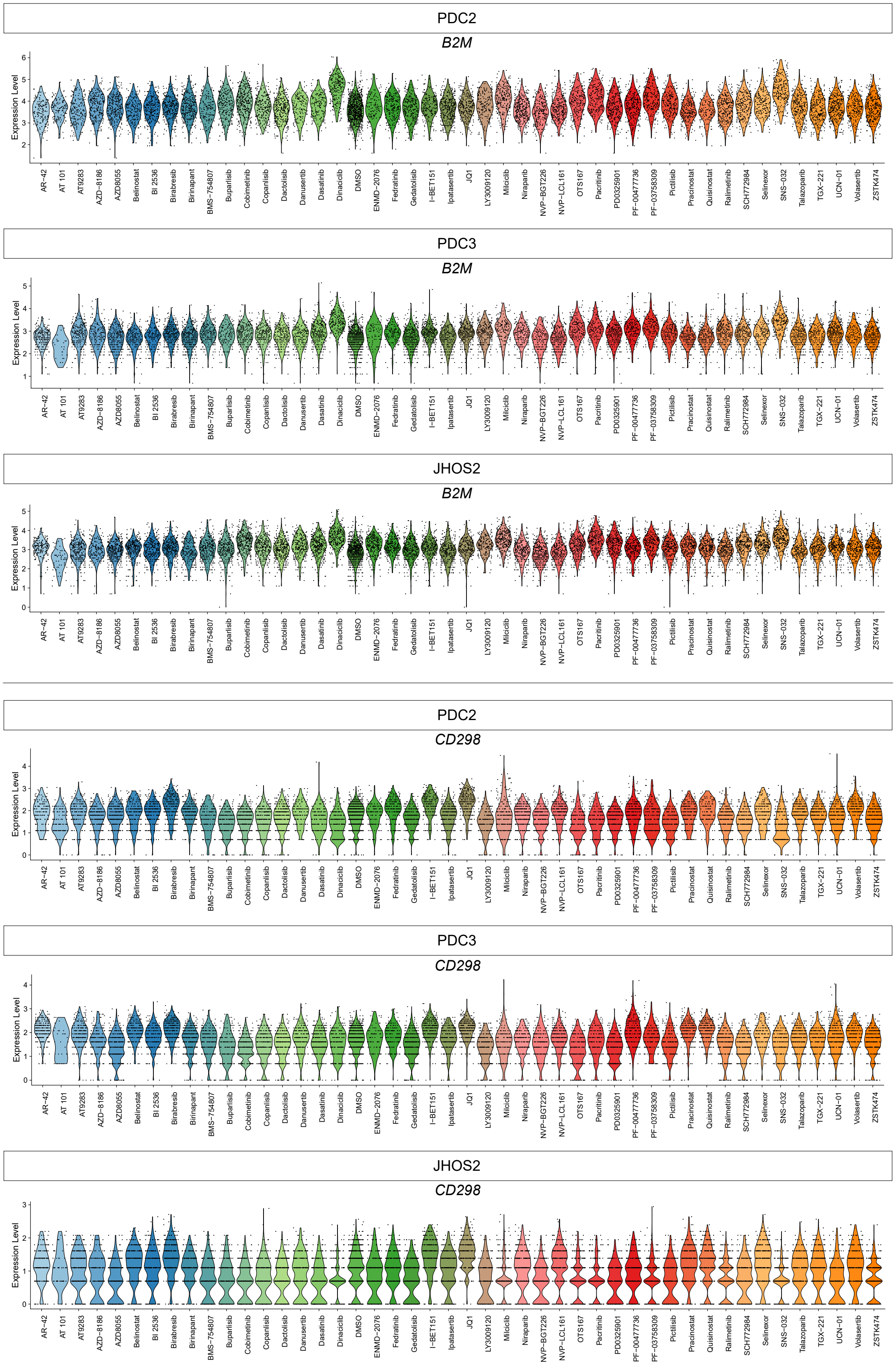

c

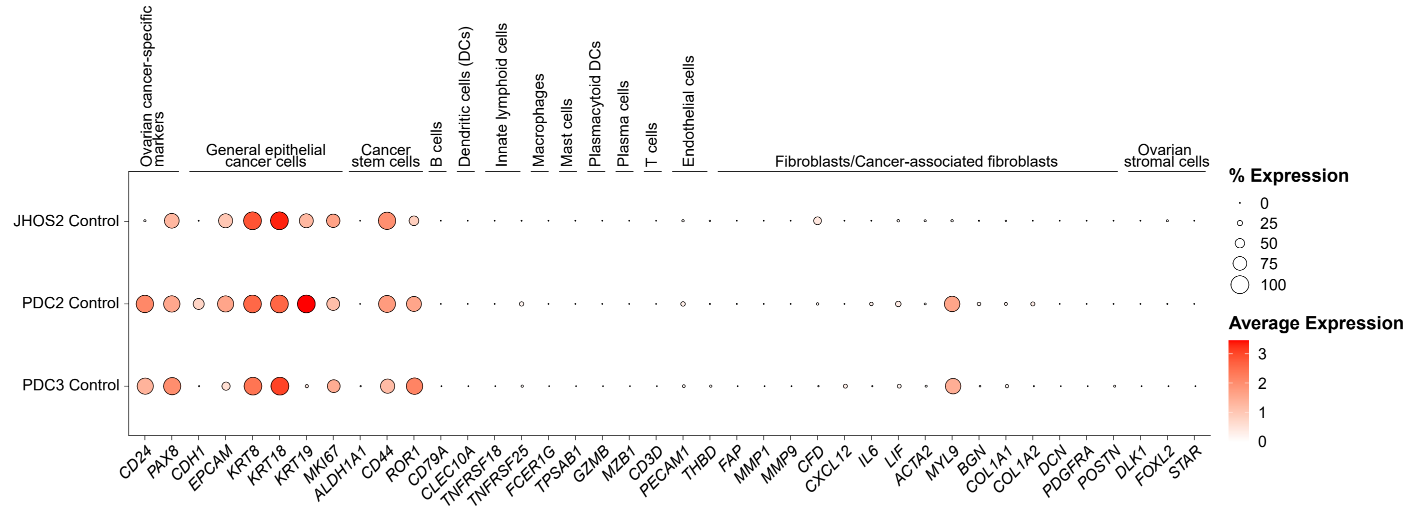

Ovarian cancer-specific markers
General epithelial cancer cells
Cancer stem cells
B cells
Dendritic cells (DCs)
Innate lymphoid cells
Macrophages
Mast cells
Plasmacytoid DCs
Plasma cells
T cells
Endothelial cells
Fibroblasts/Cancer-associated fibroblasts
Ovarian stromal cells
% Expression
0
25
50
75
100
Average Expression
3
2
1
0
JHOS2 Control
PDC2 Control
PDC3 Control
CD24
PAX8
CDH1
EPCAM
KRT8
KRT18
KRT19
MKI67
ALDH1A1
CD44
ROR1
CD79A
CLEC10A
TNFRSF18
TNFRSF25
FCER1G
TPSAB1
GZMB
MZB1
CD3D
PECAM1
THBD
FAP
MMP1
MMP9
CFD
CXCL12
IL6
LIF
ACTA2
MYL9
BGN
COL1A1
COL1A2
DCN
PDGFRA
POSTN
DLK1
FOXL2
STAR

d

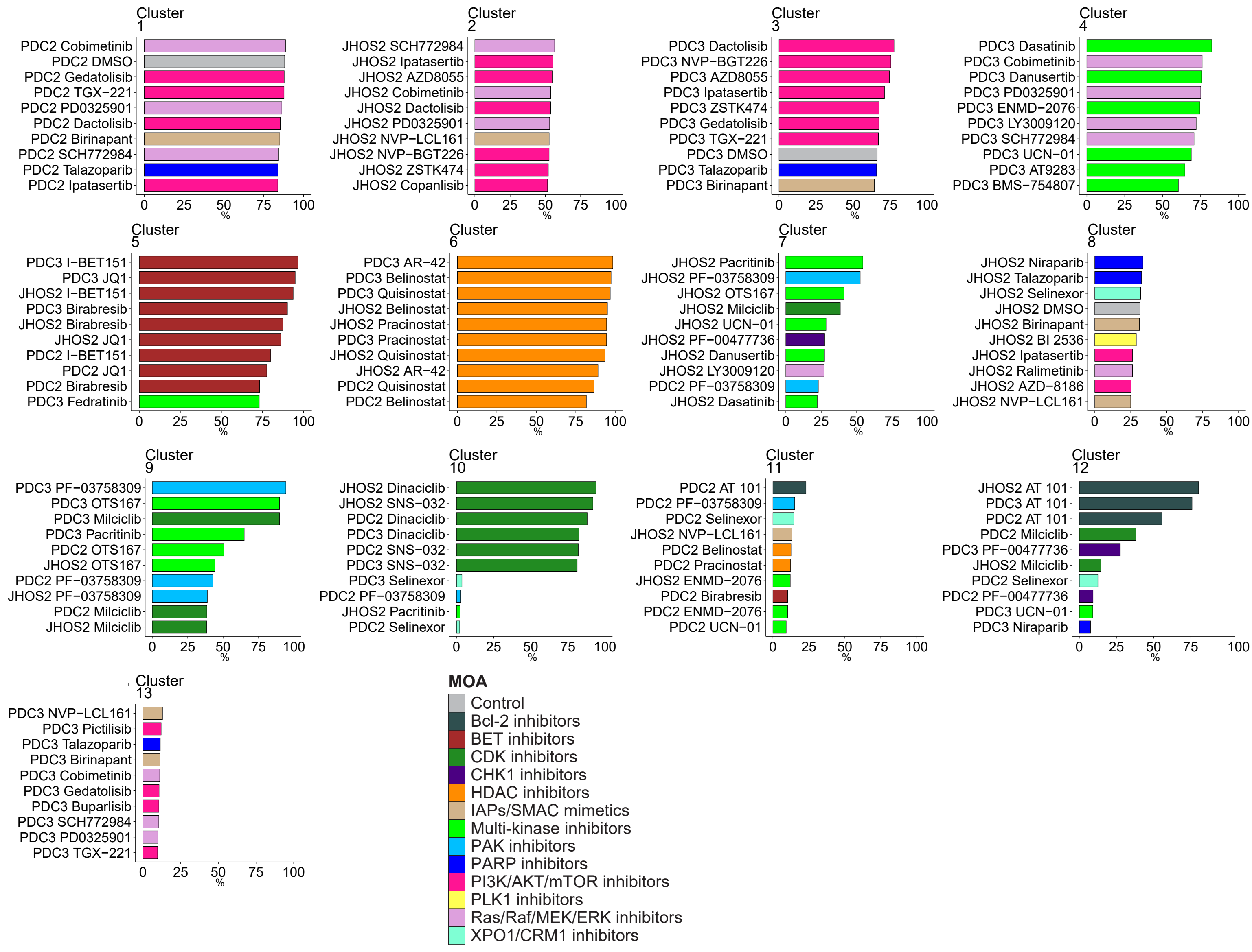

Cluster 1
PDC2 Cobimetinib
PDC2 DMSO
PDC2 Gedatolisib
PDC2 TGX−221
PDC2 PD0325901
PDC2 Dactolisib
PDC2 Birinapant
PDC2 SCH772984
PDC2 Talazoparib
PDC2 Ipatasertib
Cluster 2
JHOS2 SCH772984
JHOS2 Ipatasertib
JHOS2 AZD8055
JHOS2 Cobimetinib
JHOS2 Dactolisib
JHOS2 PD0325901
JHOS2 NVP−LCL161
JHOS2 NVP−BGT226
JHOS2 ZSTK474
JHOS2 Copanlisib
Cluster 3
PDC3 Dactolisib
PDC3 NVP−BGT226
PDC3 AZD8055
PDC3 Ipatasertib
PDC3 ZSTK474
PDC3 Gedatolisib
PDC3 TGX−221
PDC3 DMSO
PDC3 Talazoparib
PDC3 Birinapant
Cluster 4
PDC3 Dasatinib
PDC3 Cobimetinib
PDC3 Danusertib
PDC3 PD0325901
PDC3 ENMD−2076
PDC3 LY3009120
PDC3 SCH772984
PDC3 UCN−01
PDC3 AT9283
PDC3 BMS−754807
Cluster 5
PDC3 I−BET151
PDC3 JQ1
JHOS2 I−BET151
PDC3 Birabresib
JHOS2 Birabresib
JHOS2 JQ1
PDC2 I−BET151
PDC2 JQ1
PDC2 Birabresib
PDC3 Fedratinib
Cluster 6
PDC3 AR−42
PDC3 Belinostat
PDC3 Quisinostat
JHOS2 Belinostat
JHOS2 Pracinostat
PDC3 Pracinostat
JHOS2 Quisinostat
JHOS2 AR−42
PDC2 Quisinostat
PDC2 Belinostat
Cluster 7
JHOS2 Pacritinib
JHOS2 PF−03758309
JHOS2 OTS167
JHOS2 Milciclib
JHOS2 UCN−01
JHOS2 PF−00477736
JHOS2 Danusertib
JHOS2 LY3009120
PDC2 PF−03758309
JHOS2 Dasatinib
Cluster 8
JHOS2 Niraparib
JHOS2 Talazoparib
JHOS2 Selinexor
JHOS2 DMSO
JHOS2 Birinapant
JHOS2 BI 2536
JHOS2 Ipatasertib
JHOS2 Ralimetinib
JHOS2 AZD−8186
JHOS2 NVP−LCL161
Cluster 9
PDC3 PF−03758309
PDC3 OTS167
PDC3 Milciclib
PDC3 Pacritinib
PDC2 OTS167
JHOS2 OTS167
PDC2 PF−03758309
JHOS2 PF−03758309
PDC2 Milciclib
JHOS2 Milciclib
Cluster 10
JHOS2 Dinaciclib
JHOS2 SNS−032
PDC2 Dinaciclib
PDC3 Dinaciclib
PDC2 SNS−032
PDC3 SNS−032
PDC3 Selinexor
PDC2 PF−03758309
JHOS2 Pacritinib
PDC2 Selinexor
Cluster 11
PDC2 AT 101
PDC2 PF−03758309
PDC2 Selinexor
JHOS2 NVP−LCL161
PDC2 Belinostat
PDC2 Pracinostat
JHOS2 ENMD−2076
PDC2 Birabresib
PDC2 ENMD−2076
PDC2 UCN−01
Cluster 12
JHOS2 AT 101
PDC3 AT 101
PDC2 AT 101
PDC2 Milciclib
PDC3 PF−00477736
JHOS2 Milciclib
PDC2 Selinexor
PDC2 PF−00477736
PDC3 UCN−01
PDC3 Niraparib
Cluster 13
PDC3 NVP−LCL161
PDC3 Pictilisib
PDC3 Talazoparib
PDC3 Birinapant
PDC3 Cobimetinib
PDC3 Gedatolisib
PDC3 Buparlisib
PDC3 SCH772984
PDC3 PD0325901
PDC3 TGX−221
0
25
50
75
100
%
MOA
Control
Bcl-2 inhibitors
BET inhibitors
CDK inhibitors
CHK1 inhibitors
HDAC inhibitors
IAPs/SMAC mimetics
Multi-kinase inhibitors
PAK inhibitors
PARP inhibitors
PI3K/AKT/mTOR inhibitors
PLK1 inhibitors
Ras/Raf/MEK/ERK inhibitors
XPO1/CRM1 inhibitors

e

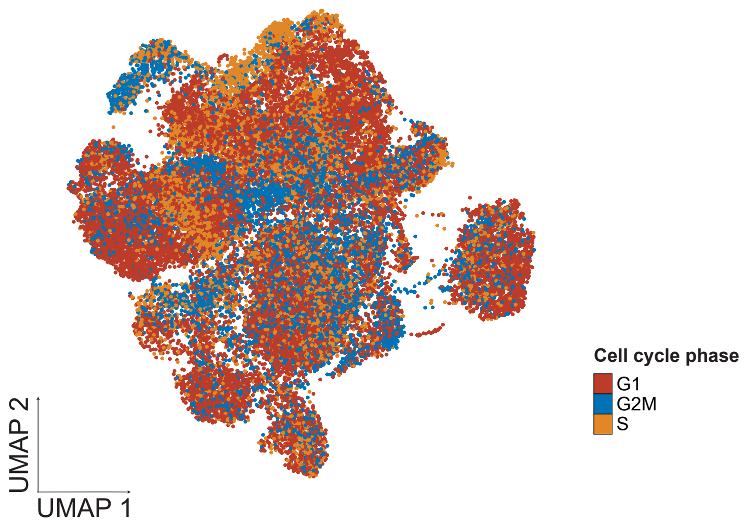

Supplementary Fig. 2: Overview of the 96-plexed scRNA-Seq output. a Heatmaps of the well-specific retrieval of high-quality cells after 96-plexed scRNA-Seq from the 3 individual batches, one for each of the examined pre-clinical model. **b** Violin plots illustrating the expression of *B2M* (top) and *CD298* (bottom) in the three models and in both control (DMSO) and cells treated with the 45 drugs included in the screening. The data is represented as distribution of the log-normalized expression data. **c** Average log-normalized scRNA-seq expression data of cell type markers within control cells from JHOS2, PDC2, and PDC3. **d** Bar plots illustrating the proportion (%) of model + drug treatment in each of the Leiden clusters. The proportion is relative to the total number of cells from a particular model that received the same compound. The filling color of the bars represents the mechanism of action (MOA) for respective compounds. For example, nearly 100% of PDC3 cells treated with AR-42 are present in Cluster 6, and most cells from PDC2, PDC3, and JHOS2 which have been treated with an HDAC inhibitor are also present in this cluster. **e** UMAP embedding of the 36,016 cells integrated from the three HGSOC pre-clinical models, colored by cell cycle phase.

Supplementary Fig. 3

a

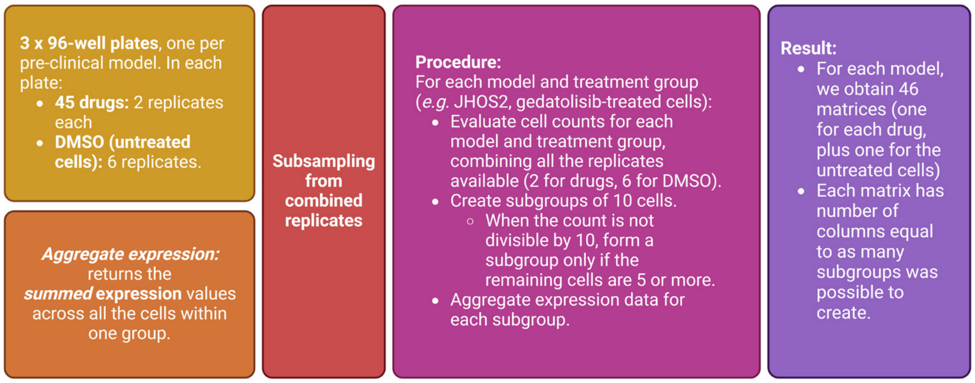

b

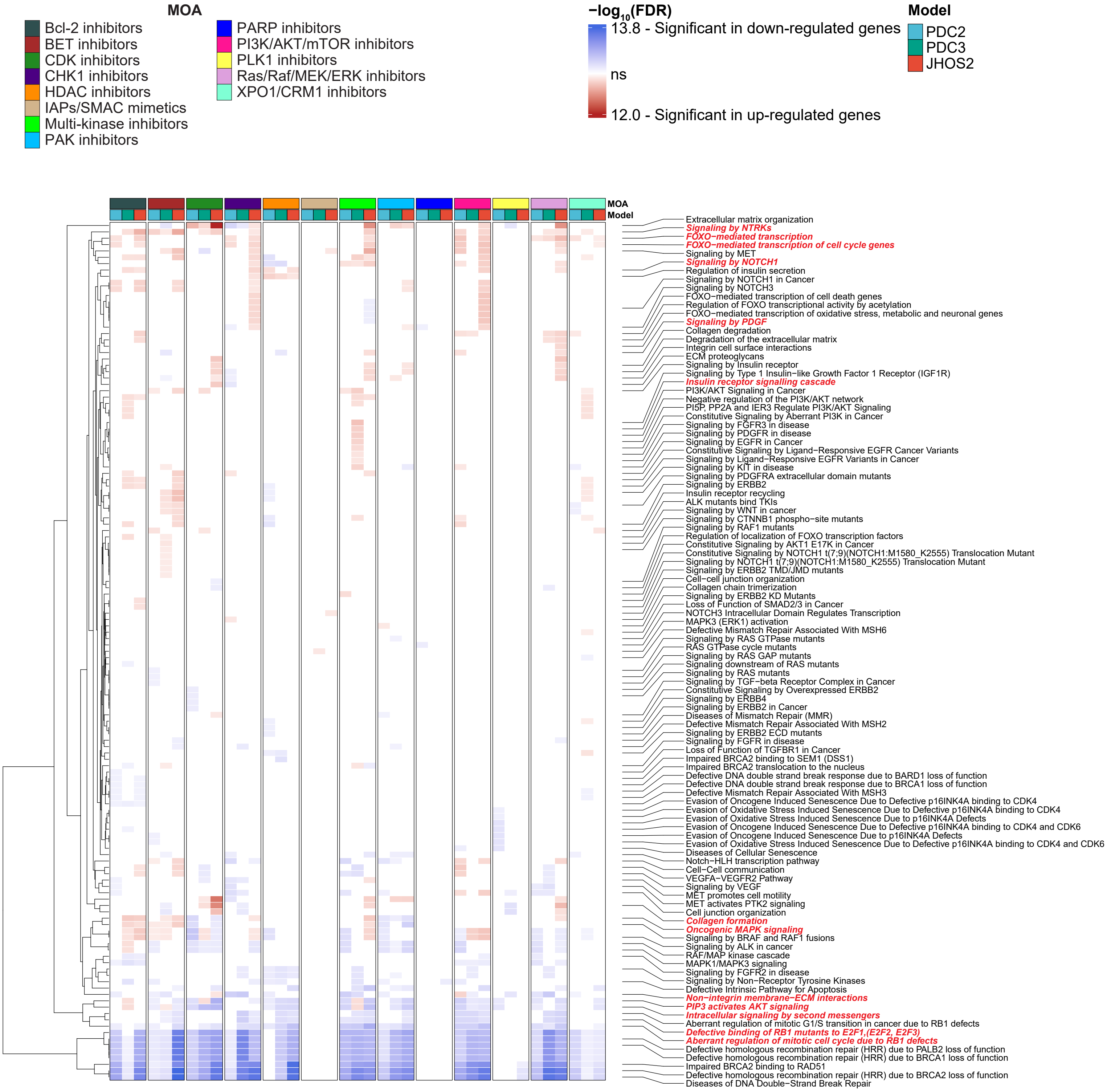

c

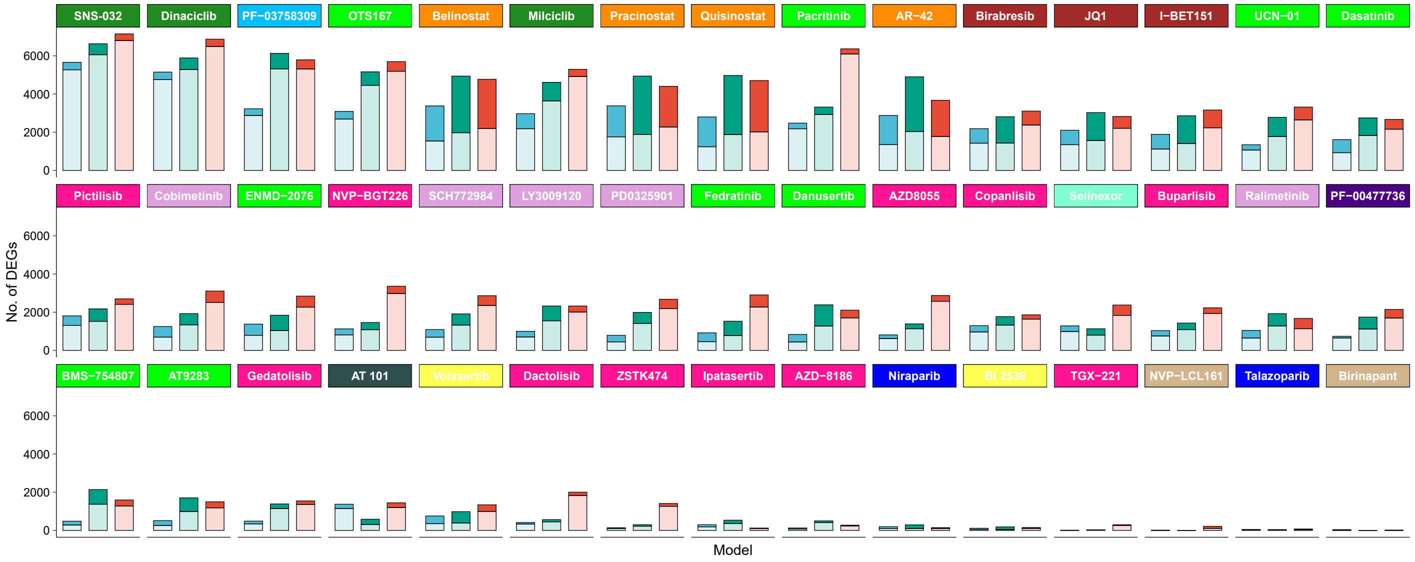

Supplementary Fig. 3: Subsample pseudobulk aggregates to mitigate small sample sizes in differential gene expression analysis. a Infographic illustrating the approach adopted to produce subsamples suitable for differential gene expression analysis. **b** Heatmap reporting the significance of the over-representation analysis results filtered for cancer development, progression, and therapeutics-related Reactome pathways. The solid color bars represent the corresponding mechanism of action (MOA, top) and the reference model (bottom). The color shading indicates the extent of the significance in over-representation among up-regulated or downregulated genes. In case of duplicated terms, and thus present as enriched among both up- and down-regulated genes, then the first and most significant instance was retained. For each MOA and model, only the top 20 terms enriched among up- and down-regulated genes were retained and filtered for the custom Reactome pathways list. **c** Benchmark of canonical scRNA-Seq method for differential gene expression analysis (Wilcoxon rank-sum test): bar charts illustrating the number of differentially expressed protein-coding genes ($|\log_2 FC| > 0.5$, $FDR < 0.01$) observed in the comparison of treated vs. untreated cells. The color-coded headers correspond to drug MOAs indicated in **b**.

Supplementary Fig. 4

a

Drug	*Substrate*
AZD-8055	ATP-competitive mTOR
AZD-8186	PI3K pan-class
Buparlisib	PI3K pan-class I
Copanlisib	ATP-competitive PI3K pan-class
Dactolisib	PI3K/mTOR dual
Gedatolisib	PI3K/mTOR dual
Ipatasertib	ATP-competitive AKT pan-class
NVP-BGT226	PI3K/mTOR dual
Pictilisib	PI3K pan-class I
TGX-221	PI3Kβ
ZSTK474	ATP-competitive PI3K pan-class

b

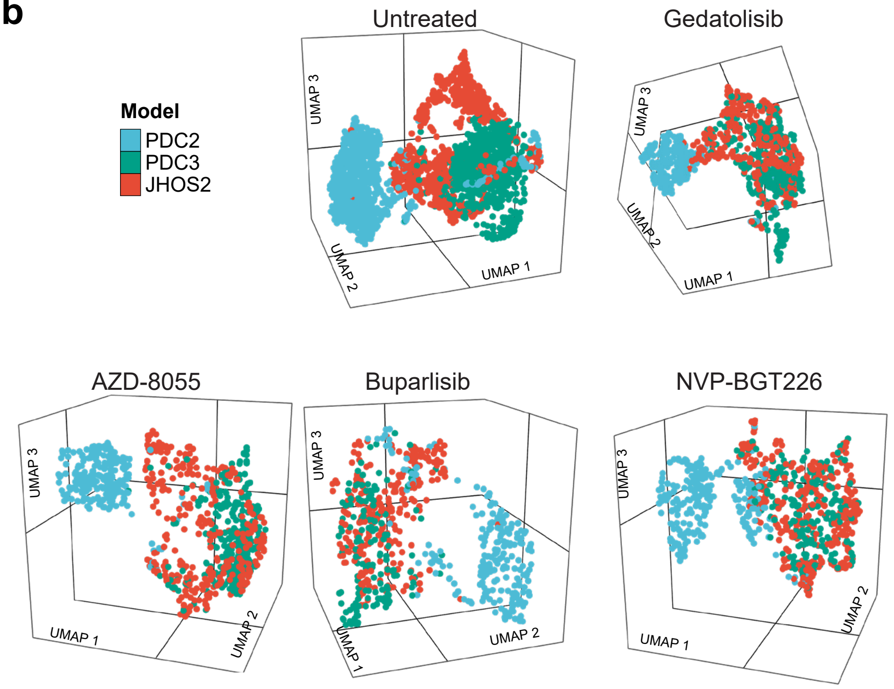

c

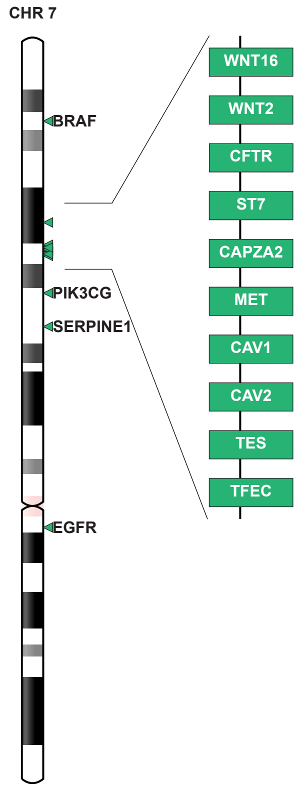

Supplementary Fig. 4: PI3K/AKT/mTOR inhibitors modulate the transcriptional regulation of caveolae-related genes and RTKs found in their genomic vicinity. a Table of the PI3K/AKT/mTOR inhibitors included in the panel for multiplexed scRNA-Seq, along with their specific target-based classification. **b** 3D UMAPs of untreated and PI3K/AKT/mTOR-inhibitor treated cells, as indicated. Colors indicate the model of origin. **c** Genomic neighborhood of *CAV1* on chromosome seven.

Supplementary Fig. 5

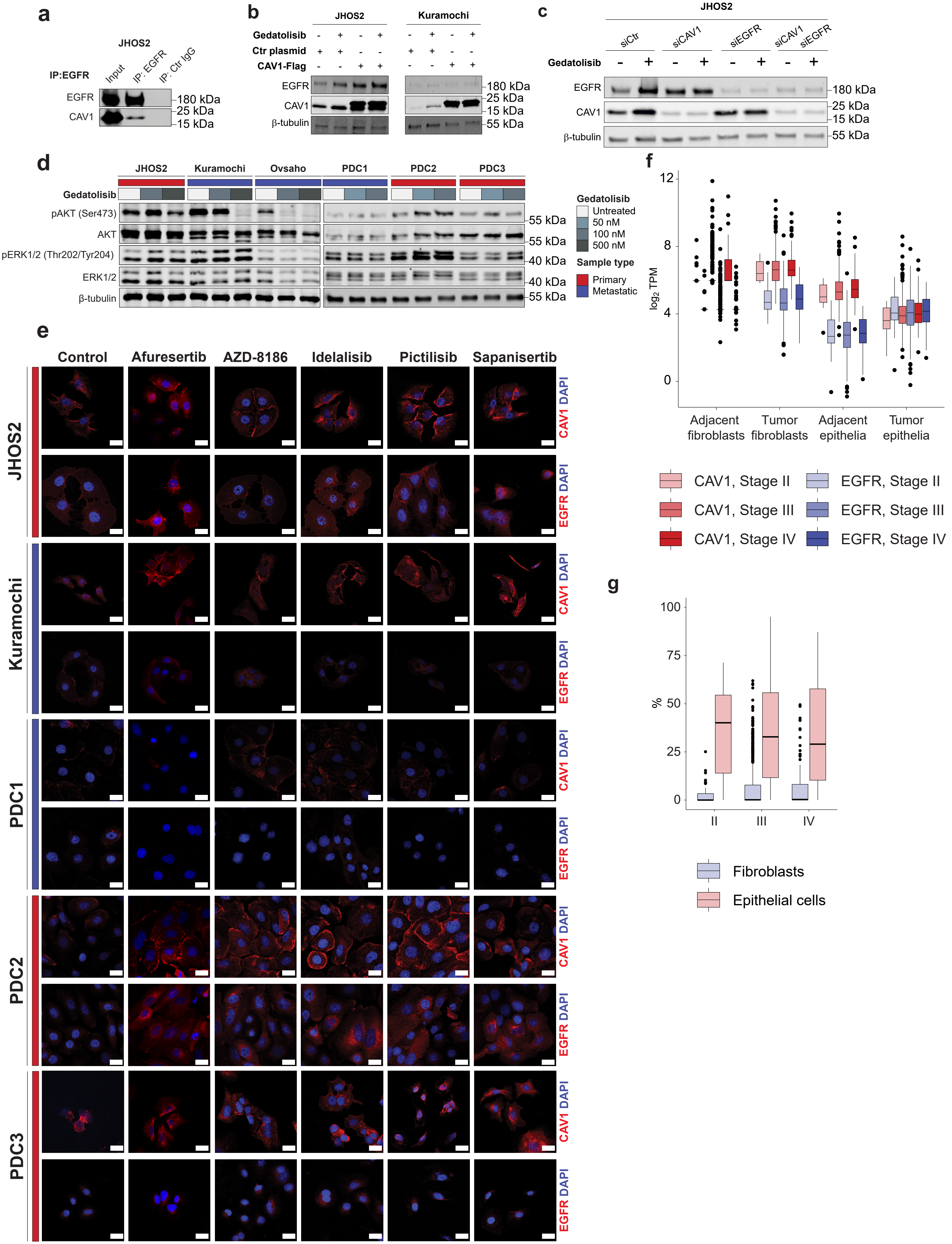

Supplementary Fig. 5: CAV1 and EGFR are modulated by PI3K/AKT/mTOR inhibitors in CAV1/EGFR expressing HGSOC. a Immunoprecipitation (IP) using EGFR antibody to assess its interaction with CAV1 in JHOS2 cells. “Input” stands for immunoblotting of input cell lysates; Ctr IgG represents the human IgG antibody used as control for IP. **b** Immunoblots of 2 HGSOC cell lines (JHOS2 and Kuramochi) with (+) and without (-) overexpression of CAV1 and treated (+) or untreated (-) with gedatolisib (concentration: 500 nM). The assay reports the protein levels of CAV1, EGFR, and β-tubulin (used as loading control). For each of the immunoblots, a representative of 4 independent experiments is shown. **c** Immunoblots of JHOS2 cells transfected with scrambled siRNA or siCtr, siCAV1, siEGFR, or combinations of thereof. The cells were treated (+) with gedatolisib (concentration: 500 nM) or left untreated (-) using equal volumes of DMSO. The assay reports the protein levels of CAV1, EGFR, and β-tubulin (used as loading control). For each of the immunoblots, a representative of 4 independent experiments is shown. ON-TARGET^{plus} smart pool of 4 siRNAs/target was included in each individual transfection. **d** Immunoblots of 8 pre-clinical models including 3 representative HGSOC cell lines (JHOS2, Kuramochi, and Ovsaho) and 5 HGSOC PDCs (PDC1–5) showing the protein levels of AKT, phospho-AKT (pAKT), ERK, phospho-ERK (pERK), and β-tubulin (used as loading control). For pAKT and pERK, the phosphorylated residue(s) are reported in parentheses. For each of the immunoblots, a representative of 4 independent experiments is shown. Color bars indicate whether a certain model is derived from primary or metastatic samples (top) or the concentration of gedatolisib used to treat the cells (bottom). **e** IF staining of 2 representative HGSOC cell lines and 3 PDCs showing CAV1 or EGFR (in red) levels, and DAPI (in blue) for nuclei. For each of the inhibitors, a concentration of 500 nM was used to treat the cells for 48 hours. The side color bars indicate whether a certain model is derived from a primary or a metastatic sample. Scale bar: 20 μm. **f** Box plots reporting the cell subtype and FIGO stage (II–IV)-specific mRNA expression of *CAV1* and *EGFR* in TCGA ovarian cancer samples (*n* = 429). The box plots illustrate (top-bottom) the five-number summary of each data group: minimum expression as log_2TPM, first (lower) quartile, median, third (upper) quartile, and maximum expression. **g** Proportion of deconvoluted epithelial (cancer) cells and fibroblasts in TCGA ovarian cancer samples analysed in **g** (*n* = 429). The box plots illustrate (top-bottom) the five-number summary of each data group: minimum proportion, first (lower) quartile, median, third (upper) quartile, and maximum percentage.

Supplementary Fig. 6

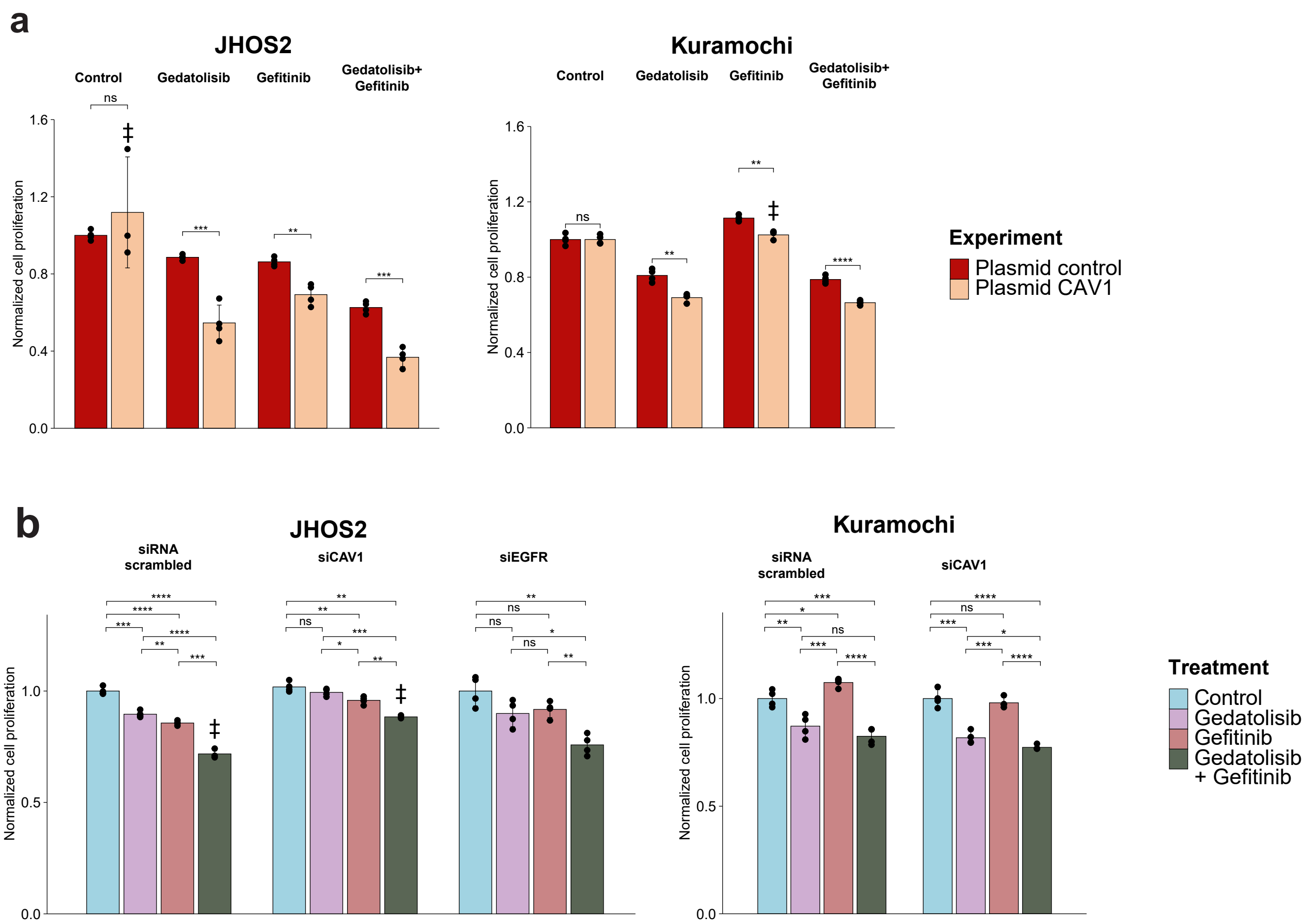

c

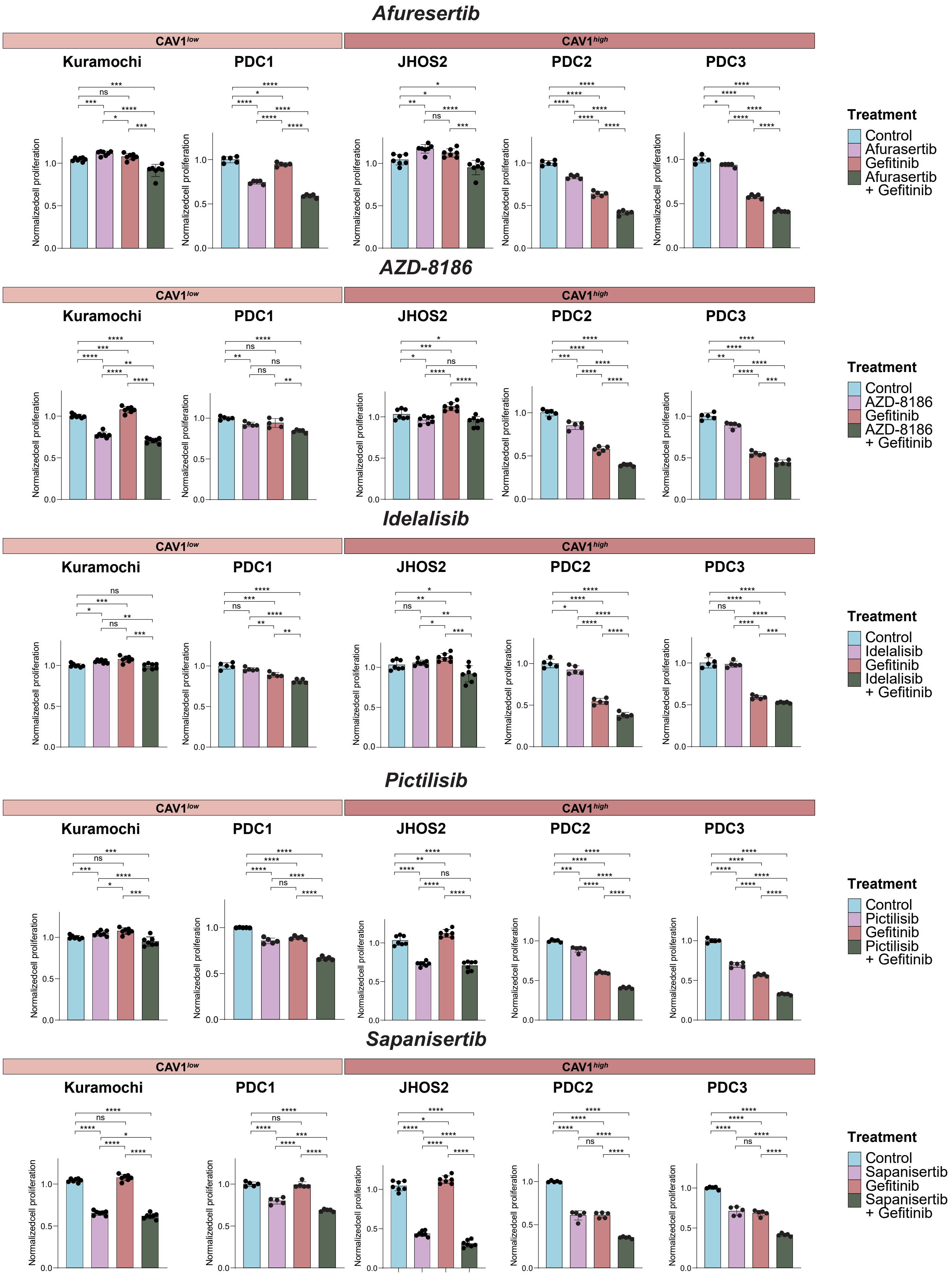
Afuresertib
CAV1^low
CAV1^high
Kuramochi
PDC1
JHOS2
PDC2
PDC3
Normalizedcell proliferation
Treatment
Control
Afurasertib
Gefitinib
Afurasertib + Gefitinib
AZD-8186
Treatment
Control
AZD-8186
Gefitinib
AZD-8186 + Gefitinib
Idelalisib
Treatment
Control
Idelalisib
Gefitinib
Idelalisib + Gefitinib
Pictilisib
Treatment
Control
Pictilisib
Gefitinib
Pictilisib + Gefitinib
Sapanisertib
Treatment
Control
Sapanisertib
Gefitinib
Sapanisertib + Gefitinib

d

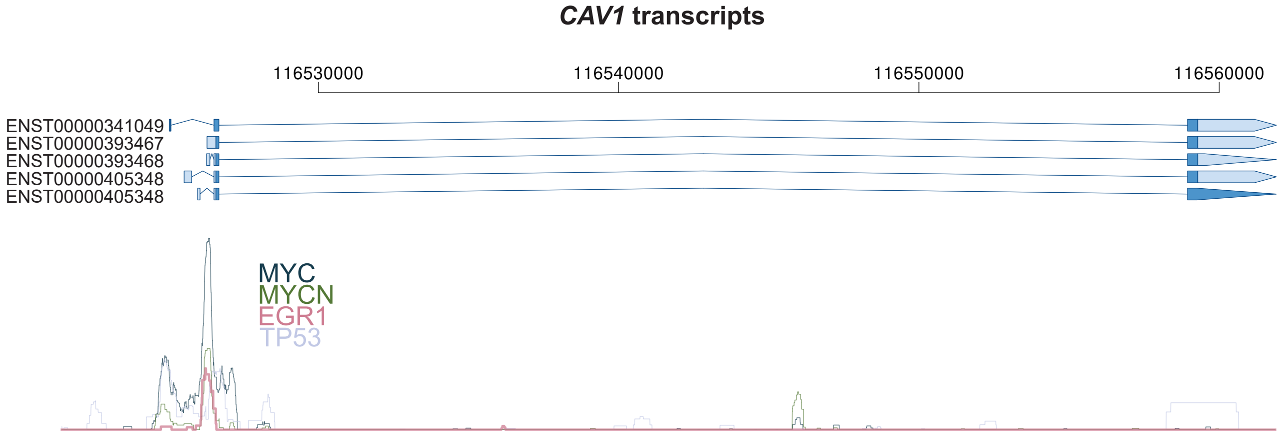

e

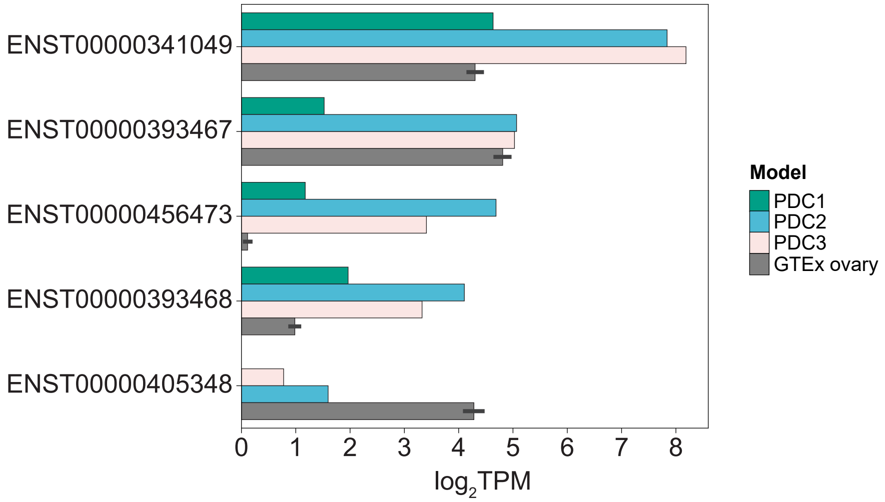

Supplementary Fig. 6: Combinatorial therapy using PI3K/AKT/mTOR inhibitors and gefitinib shows higher cytotoxicity than monotherapy in CAV1/EGFR expressing HGSOC cells. a, b Bar charts depicting the normalized cell survival of HGSOC cells where CAV1 was overexpressed *via* ectopic transfections (**b**) or either CAV1 or EGFR were knockdown *via* siRNA (**c**). The cells were either left untreated (control) or treated with indicated drugs for 72 hours. Cell survival was measured with CellTiter-Glo® assay and represented as mean ± SD of $n = 4$ ($n = 3$ for samples reporting ‡ in the respective bar) technical replicates. For the untreated samples, the average of the detected levels was set to 1 for comparison with the treated samples. The concentrations of gedatolisib and gefitinib used for JHOS2 were 500 nM and 5 µM, respectively. For Kuramochi cells, concentrations of 100 nM and 1 µM were used for gedatolisib and gefitinib, respectively. The significance of the pairwise differences between different experimental conditions was computed using the unpaired, two-tailed Student's *t*-test (ns, $p > 0.05$; *$p < 0.05$; **$p < 0.01$; ***$p < 0.001$; ****$p < 0.001$). **c** Bar charts depicting the normalized cell survival of HGSOC cells untreated (control) or treated with indicated drugs for 72 hours. Cell survival was measured with CellTiter-Glo® assay and represented as mean ± SD of $n = 5$ technical replicates for PDCs and $n = 7$ for the commercially available cell lines. For the untreated samples, the average of the detected levels was set to 1 for comparison with the treated samples. The following drugs and respective concentrations were used: afuresertib, 50 nM for JHOS2 and Kuramochi, 1 µM for PDC1–3; AZD-8186, 500 nM for JHOS2 and Kuramochi, 1 µM for PDC1–3; idelalisib, 500 nM for JHOS2 and Kuramochi, 2 µM for PDC1–3, pictilisib: 500 nM all the models; sapanisertib, 50 nM for JHOS2 and Kuramochi, 100 nM for PDC1–3. Color bars indicate the classification CAV1 protein levels verified via immunoblotting as in Figure 5a. The significance of the pairwise differences between different experimental conditions was computed using the unpaired, two-tailed Student's *t*-test (ns, $p > 0.05$; *$p < 0.05$; **$p < 0.01$; ***$p < 0.001$; ****$p < 0.0001$). **d** Gene structures of the 5 main *CAV1* isoforms. **e** Expression levels of the 5 main *CAV1* isoforms in PDC1–3 (single samples; $n = 1$) and in normal ovary tissue as extracted from GTEx. The GTEx samples are reported as mean ± SD of $n = 195$ normal ovary samples.

Uncropped Western blots related to Supplementary Figure 5a.
Black squares indicate the bands shown in Figures.

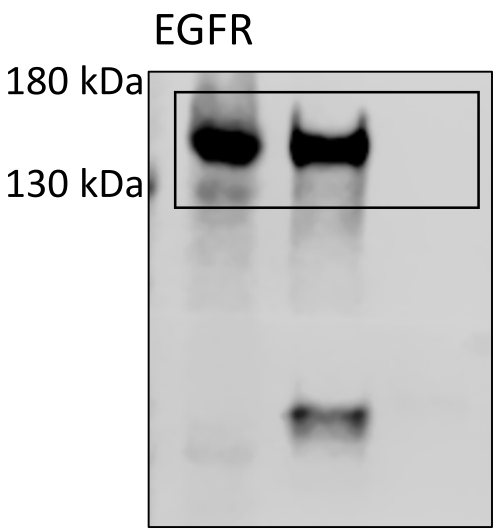

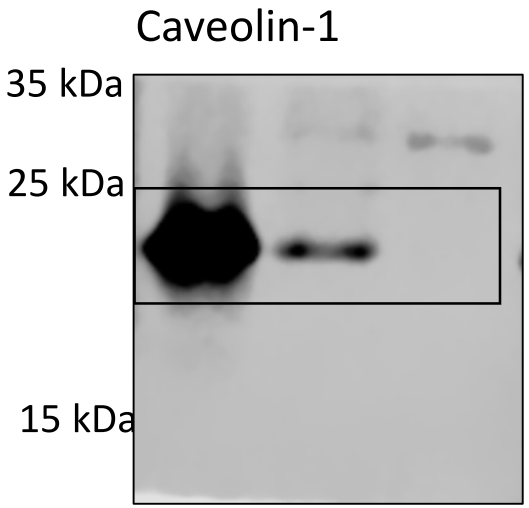

Uncropped Western blots related to Supplementary Figure 5b.
Black squares indicate the bands shown in Figures.

EGFR

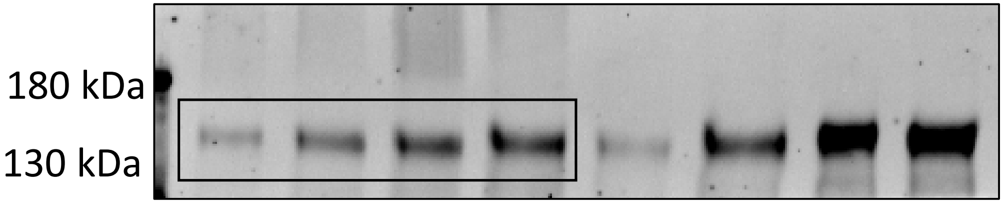

β-tubulin

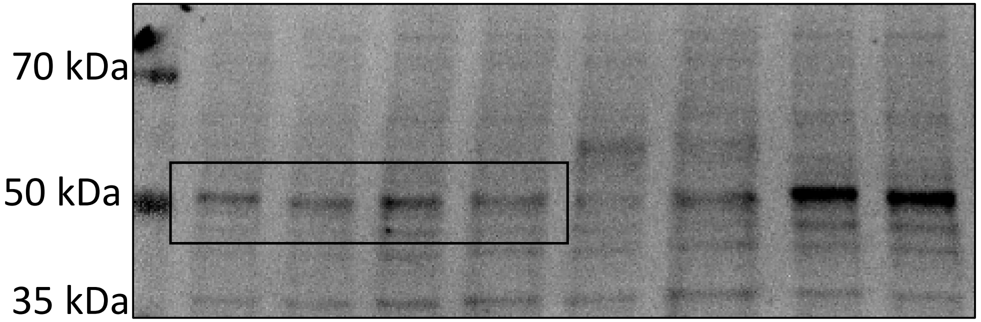

Caveolin-1

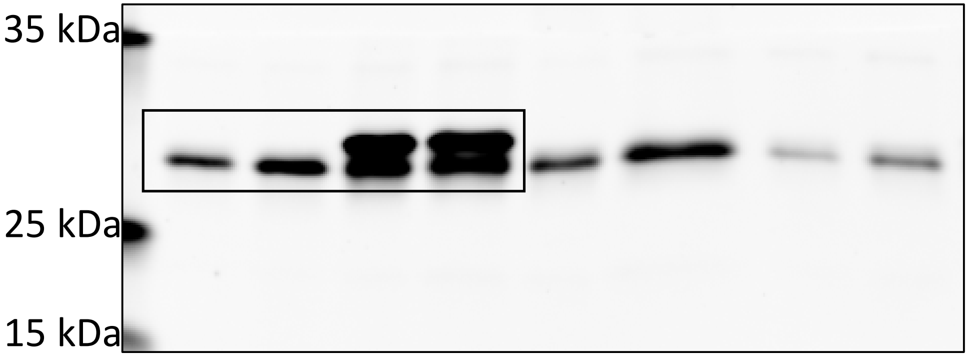

EGFR

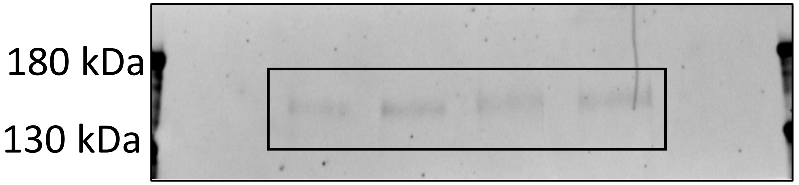

β-tubulin

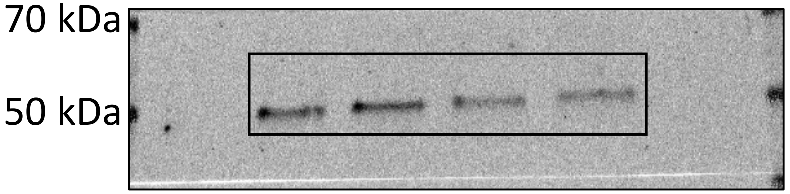

Caveolin-1

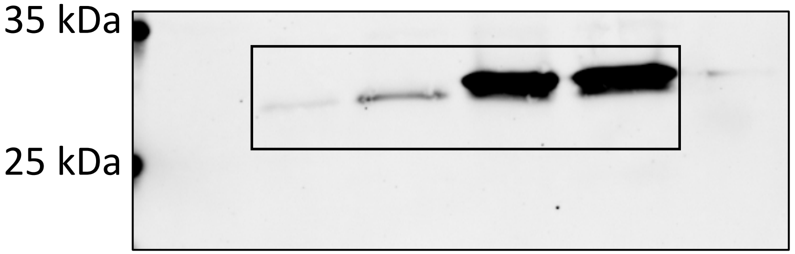

Uncropped Western blots related to Supplementary Figure 5c.
Black squares indicate the bands shown in Figures.

EGFR

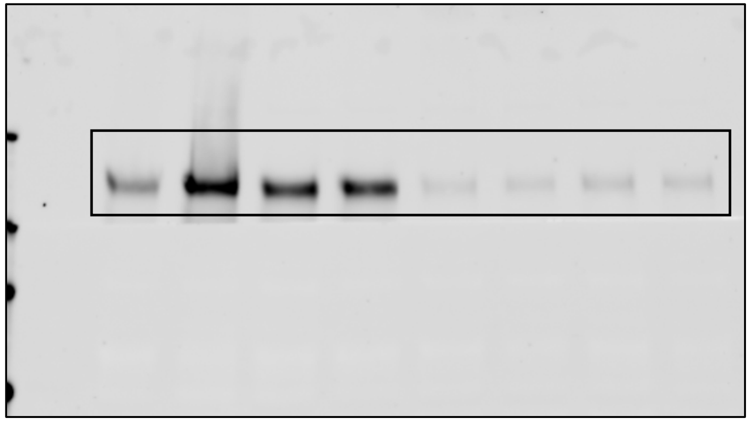

β-tubulin

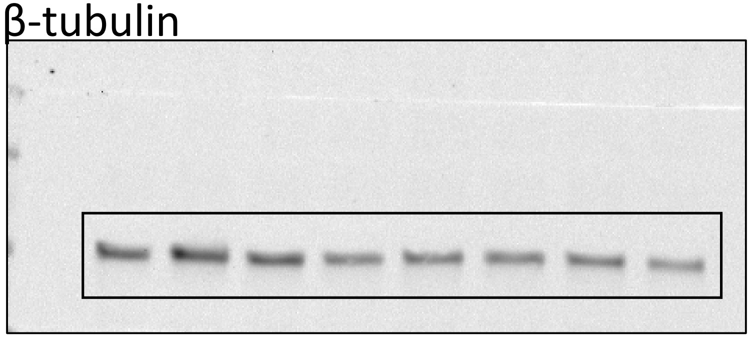

Caveolin-1

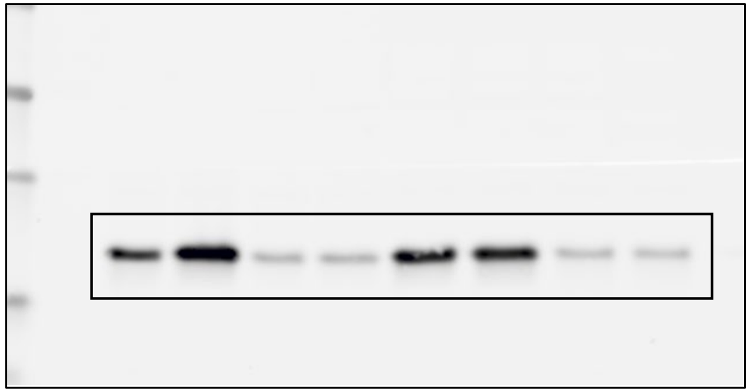

Uncropped Western blots related to Supplementary Figure 5d.
Black squares indicate the bands shown in Figures.

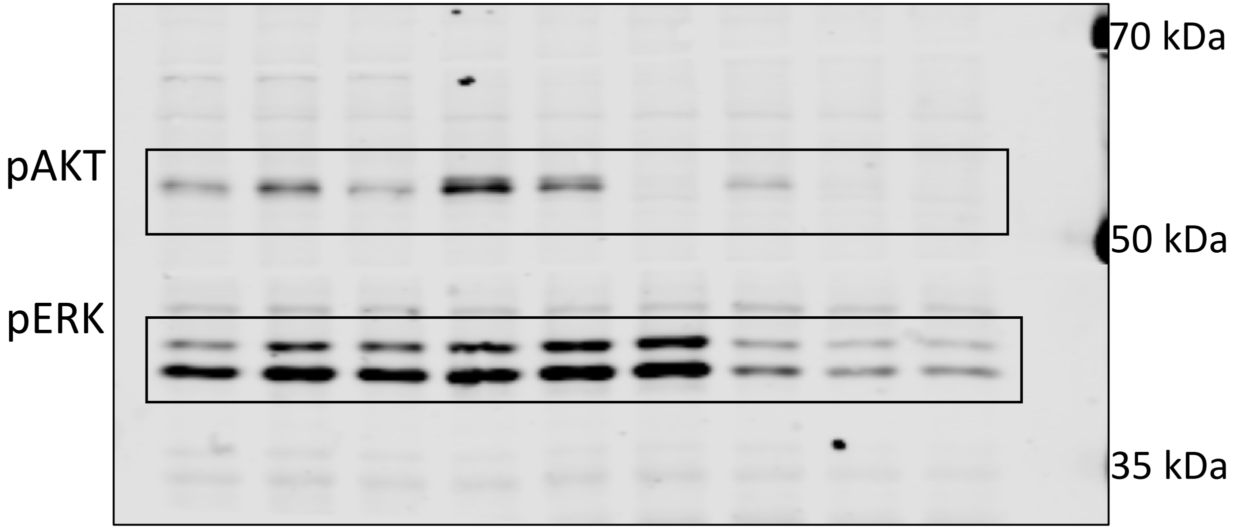

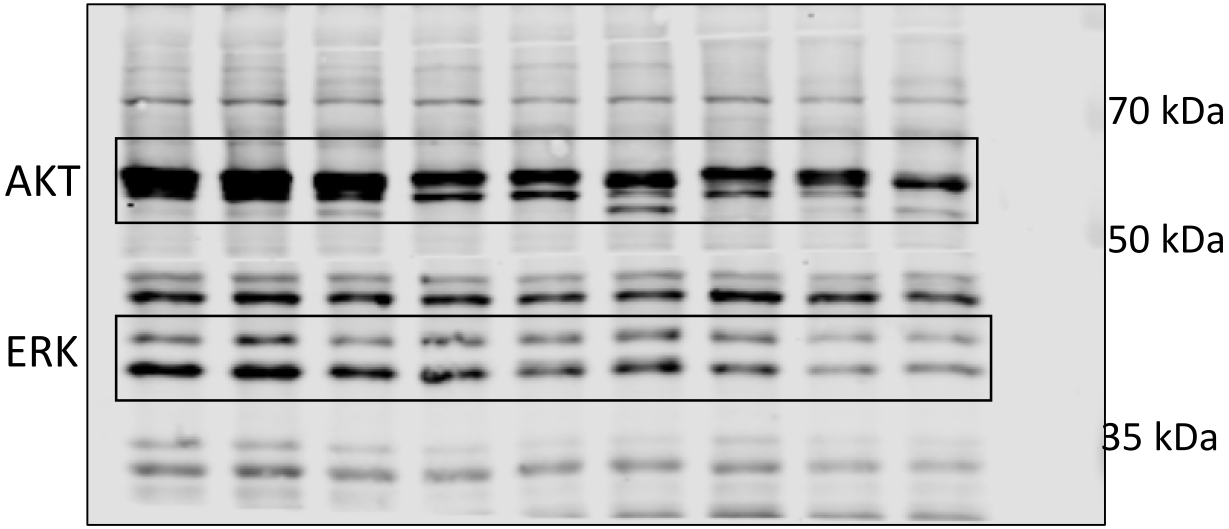

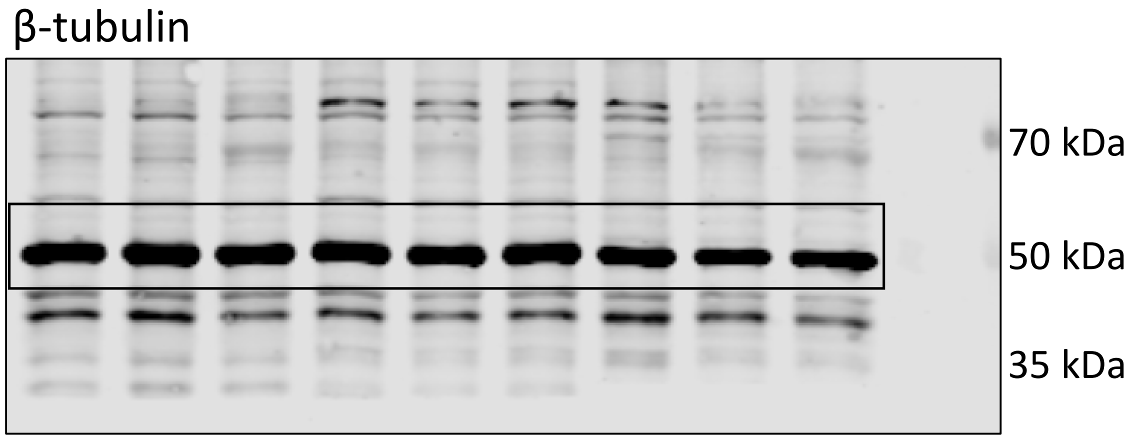

Uncropped Western blots related to Supplementary Figure 5d.
Black squares indicate the bands shown in Figure.

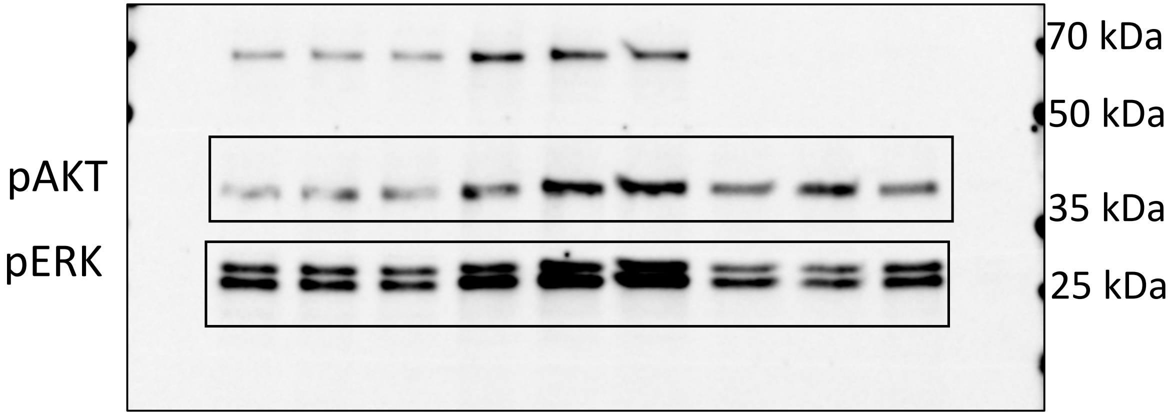

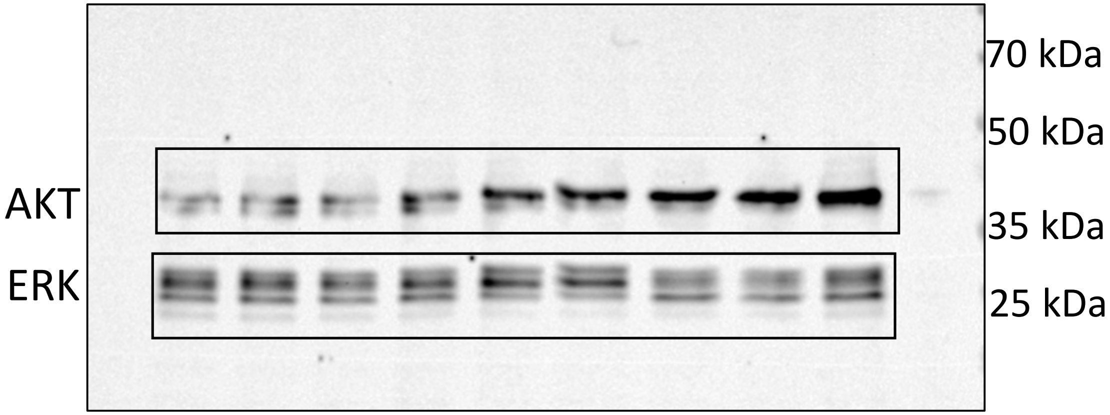

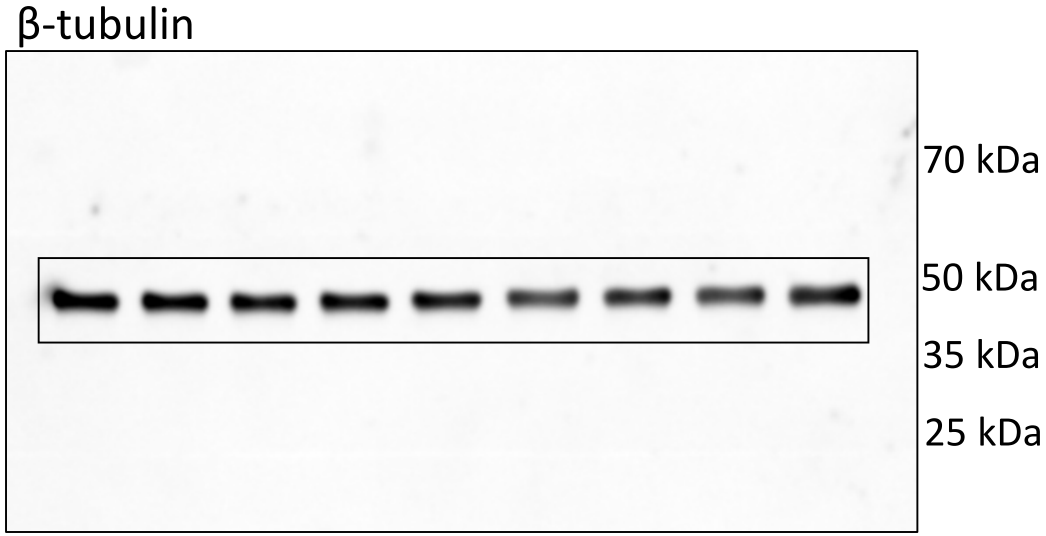

Supplement: Supplementary file 1 — Supplementary Figs. 1–6 and unprocessed scans of Supplementary Fig. 5a–d. [file 41589_2024_1761_MOESM1_ESM.pdf]
