# Supplementary material for: A multiplex single-cell RNA-Seq pharmacotranscriptomics pipeline for drug discovery
Source: Nat Chem Biol. 2024 Oct 31;21(3):432–42. doi: 10.1038/s41589-024-01761-8 (PMC11867973; doi:10.1038/s41589-024-01761-8)
Supplement: Supplementary file 2 — Reporting Summary [file 41589_2024_1761_MOESM2_ESM.pdf]

nature portfolio

Corresponding author(s): Daniela Ungureanu

Last updated by author(s): Aug 12, 2024

Reporting Summary

Nature Portfolio wishes to improve the reproducibility of the work that we publish. This form provides structure for consistency and transparency in reporting. For further information on Nature Portfolio policies, see our Editorial Policies and the Editorial Policy Checklist.

Statistics

For all statistical analyses, confirm that the following items are present in the figure legend, table legend, main text, or Methods section.

n/a	Confirmed	
☐	☒	The exact sample size (*n*) for each experimental group/condition, given as a discrete number and unit of measurement
☐	☒	A statement on whether measurements were taken from distinct samples or whether the same sample was measured repeatedly
☐	☒	The statistical test(s) used AND whether they are one- or two-sided<br>*Only common tests should be described solely by name; describe more complex techniques in the Methods section.*
☒	☐	A description of all covariates tested
☐	☒	A description of any assumptions or corrections, such as tests of normality and adjustment for multiple comparisons
☐	☒	A full description of the statistical parameters including central tendency (e.g. means) or other basic estimates (e.g. regression coefficient) AND variation (e.g. standard deviation) or associated estimates of uncertainty (e.g. confidence intervals)
☐	☒	For null hypothesis testing, the test statistic (e.g. *F*, *t*, *r*) with confidence intervals, effect sizes, degrees of freedom and *P* value noted<br>*Give P values as exact values whenever suitable.*
☒	☐	For Bayesian analysis, information on the choice of priors and Markov chain Monte Carlo settings
☒	☐	For hierarchical and complex designs, identification of the appropriate level for tests and full reporting of outcomes
☐	☒	Estimates of effect sizes (e.g. Cohen's *d*, Pearson's *r*), indicating how they were calculated

Our web collection on statistics for biologists contains articles on many of the points above.

Software and code

Policy information about availability of computer code

Data collection	Data collection was performed using 10X Genomics Chromium kit v3.1.
Data analysis	Analyses of single-cell RNA-sequencing data were performed using R 4.2.2 and computer code is available at https://github.com/ungureanulab/pharmacotranscriptomics. Specific softwares include: Cell Ranger v7.1.0; R v4.2.2; Seurat v4.3.0; SCTransform v2; Harmony v0.1.1; clustree v0.5.0; plotly v4.1.10; oppar v0.99.8; edgeR v3.40.2; gprofiler2 v0.2.1;STAR RNA-Seq aligner v2.7.10b; BayesPrism v2.0; DESeq2 v1.40; MACS2 v2; genemodel v1.10; kallisto v0.46.1; GraphPad Prism v9.3.1; Image Studio Lite (v5.2); ZEN blue Digital Imaging for Light Microscopy v3.5.093; BREEZE v1.

For manuscripts utilizing custom algorithms or software that are central to the research but not yet described in published literature, software must be made available to editors and reviewers. We strongly encourage code deposition in a community repository (e.g. GitHub). See the Nature Portfolio guidelines for submitting code & software for further information.

Data

Policy information about availability of data

All manuscripts must include a data availability statement. This statement should provide the following information, where applicable:

- Accession codes, unique identifiers, or web links for publicly available datasets
- A description of any restrictions on data availability
- For clinical datasets or third party data, please ensure that the statement adheres to our policy

Source data are provided in this paper. Publicly available datasets can be found at: GRCh38 (GENCODE v32/GENCODE v36); TCGA-OV (https://portal.gdc.cancer.gov/projects/TCGA-OV); GTEx (https://gtexportal.org/); GTRD (https://gtrd.biouml.org/); MSigDB (https://www.gsea-msigdb.org/gsea/msigdb); LINCS L1000 (https://lincsproject.org/). Source data are provided in this paper. Raw (only for JHOS2) and processed scRNA-Seq data from this manuscript have been deposited in the NCBI Genome Expression Omnibus (GEO) under the accession number GSE274905. The Leiden clusters' markers, the subsamples' pseudobulk DGE analyses results, the patient-derived bulk RNA-seq count data, and the interactive 3D UMAPs are available in Mendeley Data doi: 10.17632/j9j4mdm9yr.1. The raw FASTQ files used to generate results for the patient-derived models will be provided for scientific research upon reasonable request to fulfil privacy and ethical concerns.

Research involving human participants, their data, or biological material

Policy information about studies with human participants or human data. See also policy information about sex, gender (identity/presentation), and sexual orientation and race, ethnicity and racism.

Reporting on sex and gender	The study exclusively involved female participants, and no data pertaining to gender identity was gathered.
Reporting on race, ethnicity, or other socially relevant groupings	No reporting on race, ethnicity, or other socially relevant grouping was performed.
Population characteristics	No other population characteristic was collected and used in this study.
Recruitment	N/A
Ethics oversight	The Institutional Ethical Review Board of the University of Helsinki's Central Hospital approved the study protocol (No. 56/13/03/03/2014).

Note that full information on the approval of the study protocol must also be provided in the manuscript.

Field-specific reporting

Please select the one below that is the best fit for your research. If you are not sure, read the appropriate sections before making your selection.

☒ Life sciences ☐ Behavioural & social sciences ☐ Ecological, evolutionary & environmental sciences

For a reference copy of the document with all sections, see nature.com/documents/nr-reporting-summary-flat.pdf

Life sciences study design

All studies must disclose on these points even when the disclosure is negative.

Sample size	The sample size was determined based on our previous publication (PMIDs: 36476658; 32989221). Otherwise, the sample size was determined by TCGA, GTEx and LINCS databases.
Data exclusions	No data were excluded from the study.
Replication	Multiplexed scRNA-Seq was generated from two replicate per condition as indicated in the manuscript.<br>For cell growth assay, data was shown from three to seven replicates.<br>For the immunofluorescence assay, three or two independent technical replicate were performed and data was collected and analyzed as indicated in manuscript.<br>For immunoblotting, four independent technical replicate were performed to ensure the reproducibility.<br>For organoids' image analyses of TMRE area, three images were taken from three distinct technical replicates were included.<br>All the results from replicate for each experiment were consistent.<br>For other information, please refer to figure legends.
Randomization	For the multiplexed scRNA-seq experiments, DSRT or cell growth assay, cells (from each sample, whose identity was known) were randomly divided into the indicated groups for the drug treatment. The assignment to different treatment groups in the models undergoing multiplexed scRNA-seq was accomplished through homogeneous and random seeding of cells from a specific cell culture into a 96-well plate. Here, each well represented a distinct treatment group, with the drug being previously acoustically printed by a core facility.
Blinding	To analyze the data, knowing the conditions for sample processing was necessary for results interpretation. Data collection for scRNA-seq was done blindly.

Reporting for specific materials, systems and methods

We require information from authors about some types of materials, experimental systems and methods used in many studies. Here, indicate whether each material, system or method listed is relevant to your study. If you are not sure if a list item applies to your research, read the appropriate section before selecting a response.

Materials & experimental systems

n/a	Involved in the study
☐	☒ Antibodies
☐	☒ Eukaryotic cell lines
☒	☐ Palaeontology and archaeology
☒	☐ Animals and other organisms
☒	☐ Clinical data
☒	☐ Dual use research of concern
☒	☐ Plants

Methods

n/a	Involved in the study
☒	☐ ChIP-seq
☒	☐ Flow cytometry
☒	☐ MRI-based neuroimaging

Antibodies

Antibodies used

- Akt (pan) (40D4), Cell Signaling Technology (CST), Cat# 2920; RRID: AB_1147620 (1:1000 for immunoblotting)
- Donkey anti-Rabbit IgG (H+L) Highly Cross-Adsorbed Secondary Antibody, Alexa Fluor™ Plus 594, Thermo Fisher Scientific, Cat# A32754; RRID: AB_2762827 (1:1000 for immunofluorescence)
- AzureSpectra™ Fluorescent Secondary Antibodies Goat-anti-mouse 550, Azure Biosystems, Inc., Cat# AC2159; RRID: AB_2940961 (1:15000 for immunoblotting)
- Caveolin-1 (D46G3), Cell Signaling Technology (CST), Cat# 3267; RRID: AB_2275453 (1:1000 for immunoblotting, 1:500 for immunofluorescence)
- CD298, BioLegend, Cat# 341712; RRID: AB_2876646 (300 µg for Cell Hashing)
- EGFR (D38B1), Cell Signaling Technology (CST), Cat# 4267; RRID: AB_2246311 (1:1000 for immunoblotting, 1:500 for immunofluorescence)
- ERK1/2 (L34F12), Cell Signaling Technology (CST), Cat# 4696; RRID: AB_390780 (1:1000 for immunoblotting)
- IRDye® 680RD Donkey anti-Rabbit IgG, LI-COR, Cat# 926-68073; RRID: AB_10954442 (1:15000 for immunoblotting)
- IRDye® 800CW Donkey anti-Mouse IgG, LI-COR, Cat# 926-32212; RRID: AB_621847 (1:15000 for immunoblotting)
- pAKT (Ser473) (D9E), Cell Signaling Technology (CST), Cat# 4060; RRID: AB_2315049 (1:1000 for immunoblotting)
- pERK (Thr202/Tyr204), Cell Signaling Technology (CST), Cat# 9101; RRID: AB_331646 (1:1000 for immunoblotting)
- β2microglobulin, BioLegend, Cat# 316302; RRID: AB_492835 (300 µg for Cell Hashing)
- β-tubulin (D3U1W), Cell Signaling Technology (CST), Cat# 86298; RRID: AB_2715541 (1:1000 for immunoblotting)

Validation

All the antibodies used are commercially available and their validation statements are available on the manufacturer's website:
- Akt (pan)
https://www.cellsignal.com/products/primary-antibodies/akt-pan-40d4-mouse-mab/2920

- Donkey anti-Rabbit IgG (H+L) Highly Cross-Adsorbed Secondary Antibody, Alexa Fluor™ Plus 594
https://www.thermofisher.com/antibody/product/Donkey-anti-Rabbit-IgG-H-L-Highly-Cross-Adsorbed-Secondary-Antibody-Polyclonal/A3275

- AzureSpectra™ Fluorescent Secondary Antibodies Goat-anti-mouse 550
https://azurebiosystems.com/product/azurespectra-fluorescent-secondary-antibodies/

- Caveolin-1 (D46G3)
https://www.cellsignal.com/products/primary-antibodies/caveolin-1-d46g3-xp-rabbit-mab/3267

- CD298
https://www.biolegend.com/en-us/products/purified-anti-human-cd298-antibody-19971

- EGFR (D38B1)
https://www.cellsignal.com/products/primary-antibodies/egf-receptor-d38b1-xp-rabbit-mab/4267

- ERK1/2 (L34F12)
https://www.cellsignal.com/products/primary-antibodies/p44-42-mapk-erk1-2-l34f12-mouse-mab/4696

- IRDye® 680RD Donkey anti-Rabbit IgG
https://www.licor.com/bio/reagents/irdye-680rd-donkey-anti-rabbit-igg-secondary-antibody

- IRDye® 800CW Donkey anti-Mouse IgG
https://www.licor.com/bio/reagents/irdye-800CW-donkey-anti-mouse-igg-secondary-antibody

- pAKT (Ser473) (D9E)
https://www.cellsignal.com/products/primary-antibodies/phospho-akt-ser473-d9e-xp-rabbit-mab/4060

- pERK (Thr202/Tyr204)
https://www.cellsignal.com/products/primary-antibodies/phospho-p44-42-mapk-erk1-2-thr202-tyr204-antibody/9101

- β2microglobulin
https://www.biolegend.com/en-us/products/purified-anti-human-beta2-microglobulin-antibody-3078

- β-tubulin
https://www.cellsignal.com/products/primary-antibodies/b-tubulin-d3u1w-mouse-mab/86298

Eukaryotic cell lines

Policy information about cell lines and Sex and Gender in Research

Cell line source(s)

- JHOS2, Riken BioResource Research Center (BRC), RRID: CVCL_4647
- Kuramochi, Japanese Collection of Research Bioresources (JCRB), RRID: CVCL_1345
- Ovsaho, Japanese Collection of Research Bioresources (JCRB), RRID: CVCL_3114

Authentication

The cell lines were authenticated on June 22nd, 2023. Specifically, Genomics Unit of Technology Centre, Institute for Molecular Medicine Finland (FIMM), has authenticated JHOS2, Kuramochi and Ovsaho with the Promega GenePrint24 System. The Promega GenePrint24 system allows co-amplification and detection of 24 human loci (22 autosomal) STR loci and Amelogenin and DYS391 for gender identification). These loci collectively provide a genetic profile with a random match probability of 1 in 2.92 x 109
for 10 markers, and even lower for all 24. The results obtained were compared to results found in ATCC STR database of Human Cell Lines, JCRB STR database of Human Cell Lines, ICLC STR database of Human Cell Lines (CLIMA2.1 search available at http://bioinformatics.hsanmartino.it/clima2/) and the DSMZ Online STR database (http://cell.dive.dsmz.de/str) as well as the Cellosaurus resource (https://web.cellosaurus.org). The identity estimates are calculated according to the allele information found from these sources.

- JHOS2
20/20 expected and detected alleles were identical, giving an overall identity estimate of 100 % (Tanabe algorithm). Based on these comparisons JHOS2 parental can be confirmed to be identical to JHOS-2 cell line (CVCL_4647) (>80% allelic identity needed for confirmation according to the International Cell Line Authentication Committee (ICLAC) guidelines).

- Kuramochi
30/32 expected and detected alleles were identical, giving an overall identity estimate of 93.8 % (Tanabe algorithm). Based on these comparisons Kuramochi parental can be confirmed to be identical to Kuramochi cell line (CVCL_1345) (>80% allelic identity needed for confirmation according to the International Cell Line Authentication Committee (ICLAC) guidelines).

- Ovsaho
31/32 expected and detected alleles were identical, giving an overall identity estimate of 96.9 % (Tanabe algorithm). Based on these comparisons Ovsaho parental can be confirmed to be identical to Ovsaho cell line (CVCL_3114) (>80% allelic identity needed for confirmation according to the International Cell Line Authentication Committee (ICLAC) guidelines).

Mycoplasma contamination

All the cell lines used in the study tested negative for Mycoplasma.

Commonly misidentified lines
(See ICLAC register)

No commonly misidentified lines were used.
