# Supplementary material for: A multiplex single-cell RNA-Seq pharmacotranscriptomics pipeline for drug discovery
Source: Nat Chem Biol. 2024 Oct 31;21(3):432–42. doi: 10.1038/s41589-024-01761-8 (PMC11867973; doi:10.1038/s41589-024-01761-8)

Uncropped Western blots related to Figure 5a.
Black squares indicate the bands shown in Figure.

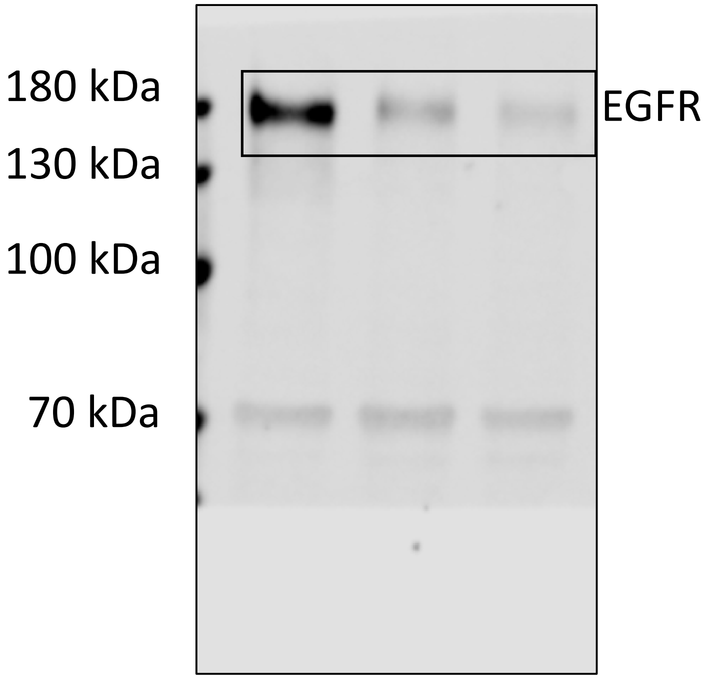

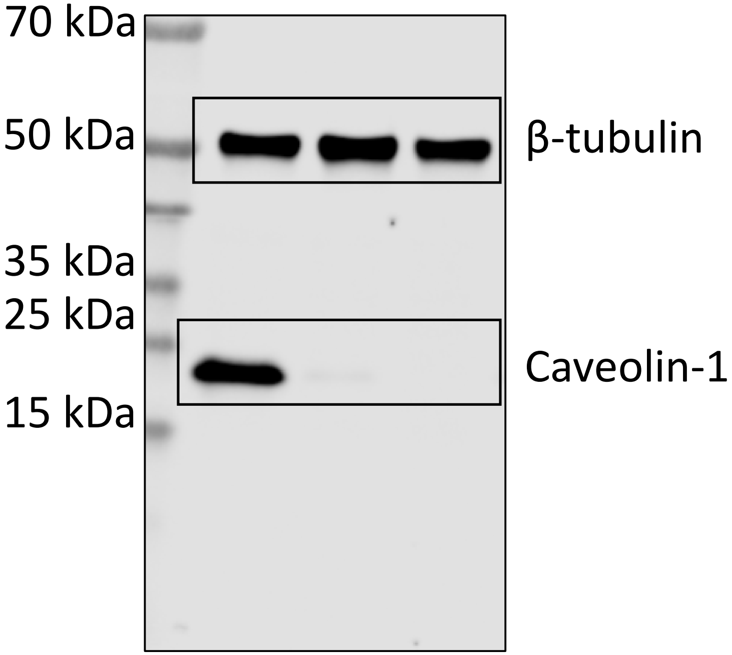

Uncropped Western blots related to Figure 5a.
Black squares indicate the bands shown in Figures.

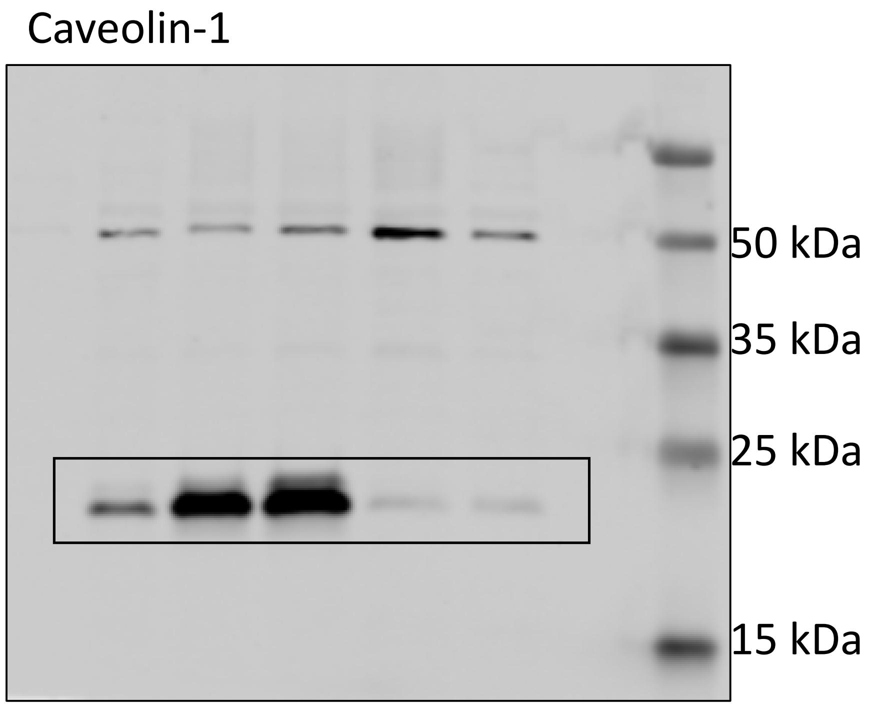

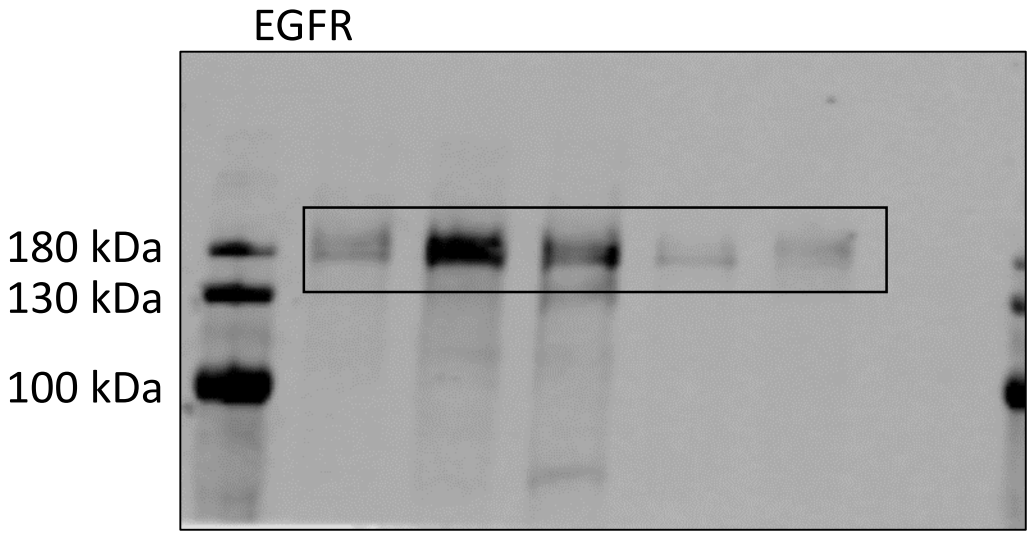

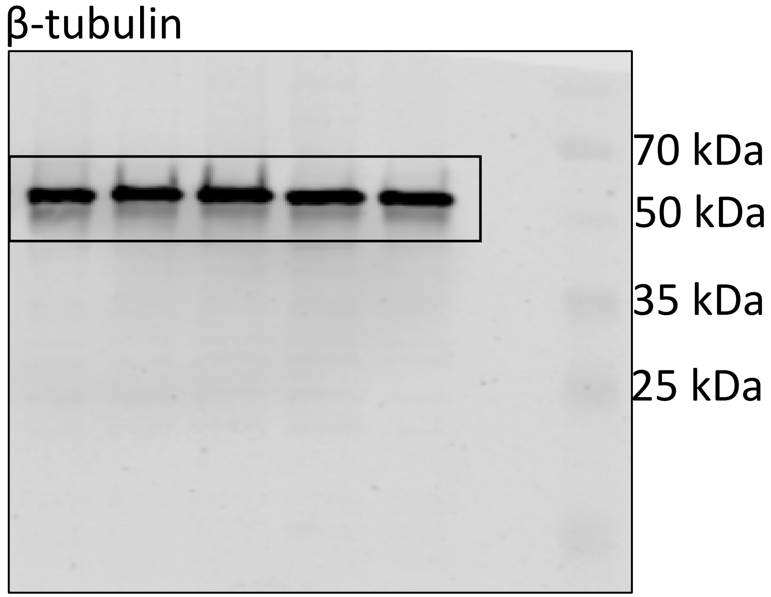

Uncropped Western blots related to Figure 5b.
Black squares indicate the bands shown in Figures.

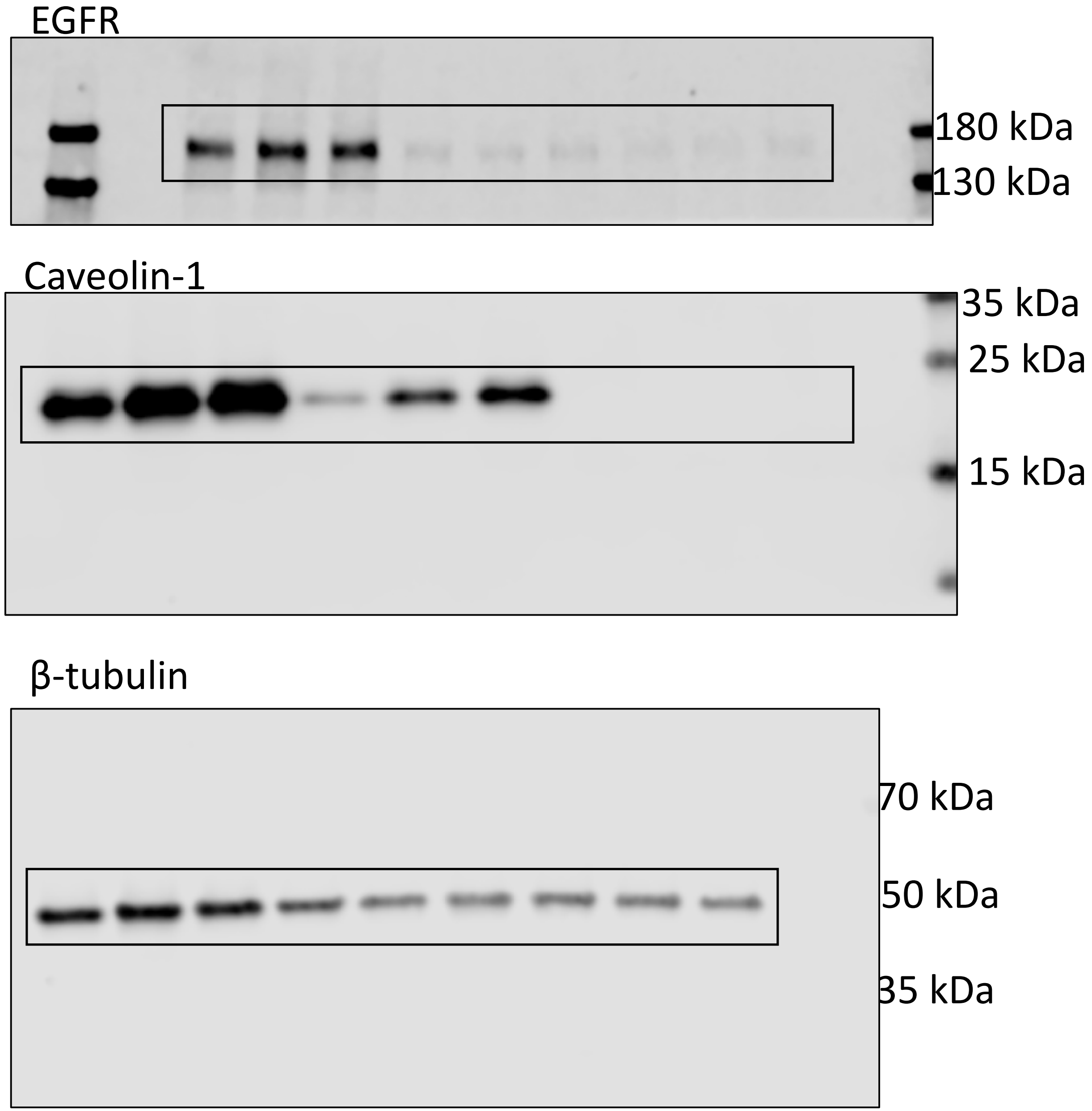

Uncropped Western blots related to Figure 5c.
Black squares indicate the bands shown in Figures.

EGFR

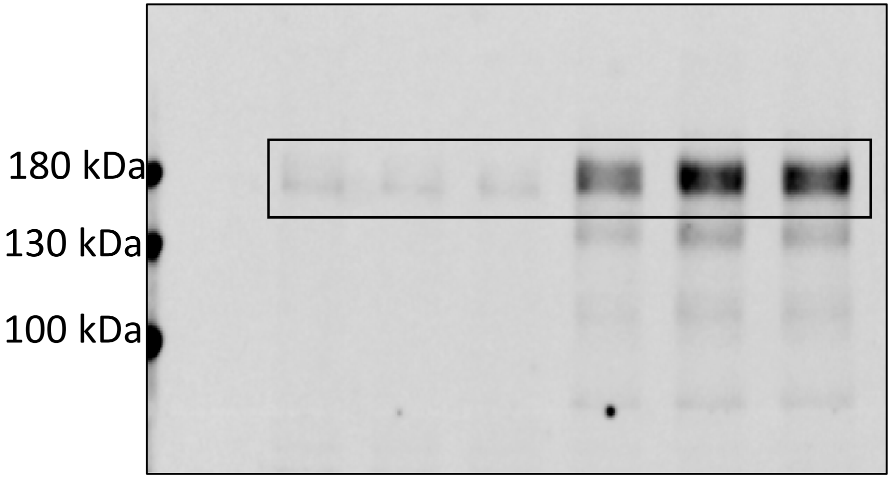

EGFR

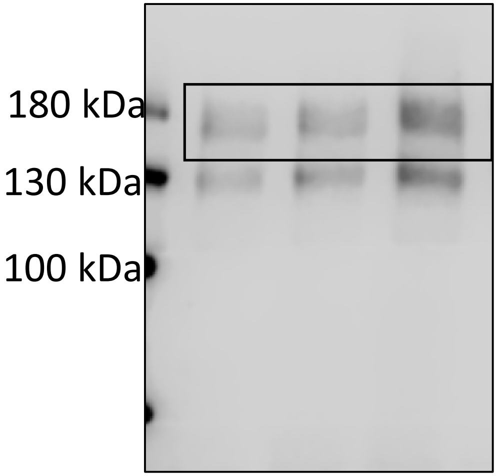

Caveolin-1

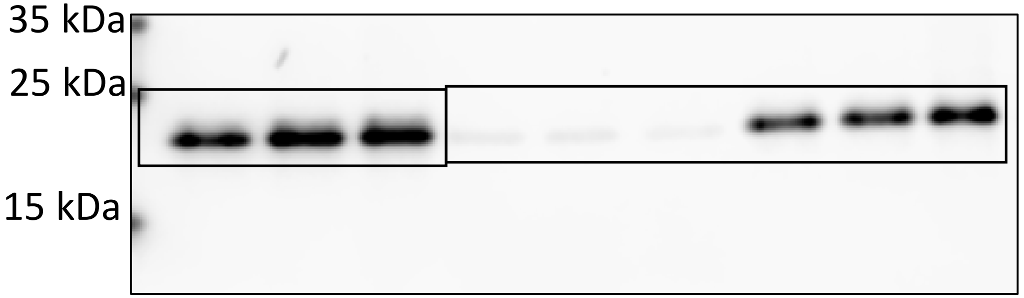

β-tubulin

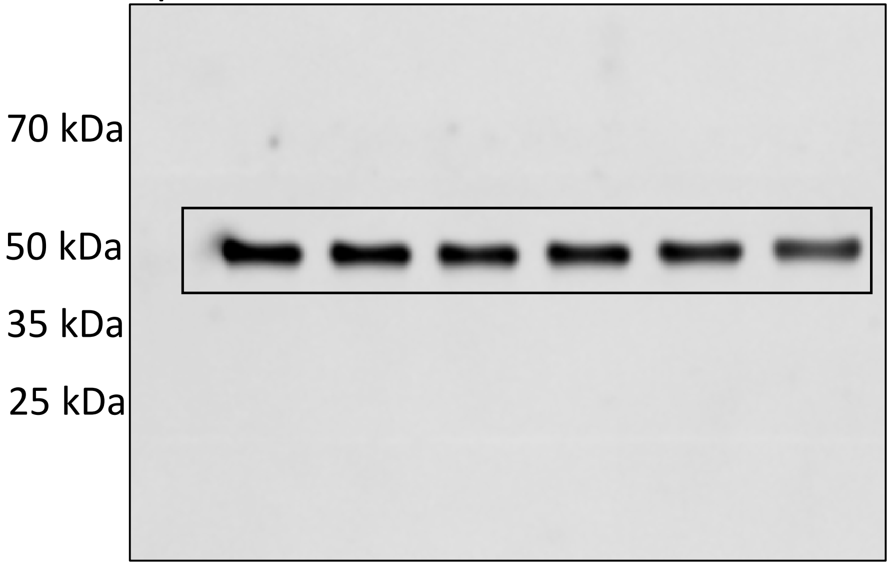

β-tubulin

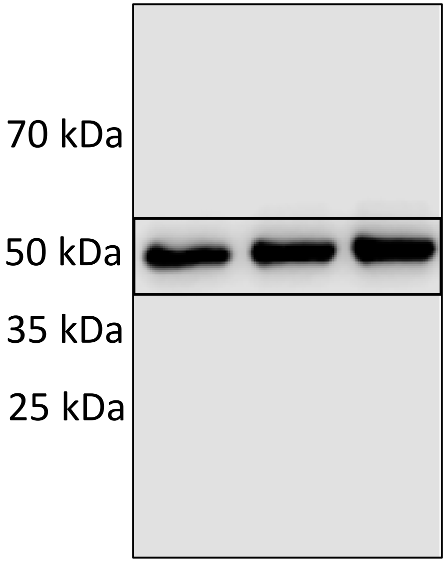

Supplement: Supplementary file 7 — Unprocessed western blots. [file 41589_2024_1761_MOESM7_ESM.pdf]
